# Ciliary membrane proteins traffic through the Golgi via a Rabep1/GGA1/Arl3-dependent mechanism

**DOI:** 10.1038/ncomms6482

**Published:** 2014-11-18

**Authors:** Hyunho Kim, Hangxue Xu, Qin Yao, Weizhe Li, Qiong Huang, Patricia Outeda, Valeriu Cebotaru, Marco Chiaravalli, Alessandra Boletta, Klaus Piontek, Gregory G. Germino, Edward J. Weinman, Terry Watnick, Feng Qian

**Affiliations:** 1 Division of Nephrology, Department of Medicine, University of Maryland School of Medicine, Baltimore, Maryland 21201, USA; 2 Department of Medicine, The Johns Hopkins University School of Medicine, Baltimore, Maryland 21205, USA; 3 Division of Genetics and Cell Biology, San Raffaele Scientific Institute, 20132 Milan, Italy; 4 National Institute of Diabetes and Digestive and Kidney Disease, National Institute of Health, Bethesda, Maryland 20892, USA

## Abstract

Primary cilia contain specific receptors and channel proteins that sense the extracellular milieu. Defective ciliary function causes ciliopathies such as autosomal dominant polycystic kidney disease (ADPKD). However, little is known about how large ciliary transmembrane proteins traffic to the cilia. Polycystin-1 (PC1) and -2 (PC2), the two ADPKD gene products, are large transmembrane proteins that co-localize to cilia where they act to control proper tubular diameter. Here we describe that PC1 and PC2 must interact and form a complex to reach the *trans*-Golgi network (TGN) for subsequent ciliary targeting. PC1 must also be proteolytically cleaved at a GPS site for this to occur. Using yeast two-hybrid screening coupled with a candidate approach, we identify a Rabep1/GGA1/Arl3-dependent ciliary targeting mechanism, whereby Rabep1 couples the polycystin complex to a GGA1/Arl3-based ciliary trafficking module at the TGN. This study provides novel insights into the ciliary trafficking mechanism of membrane proteins.

## Abstract (web summary)

Polycystins (PC) 1 and 2 are large transmembrane proteins that play a vital role in the function of primary cilia. Here, Kim *et al.* identify the requirements for polycystin trafficking to the cilium, involving a PC1–PC2 interaction, PC1 proteolytic cleavage and a specific trafficking module at the *trans*-Golgi network.

## Graphical abstract

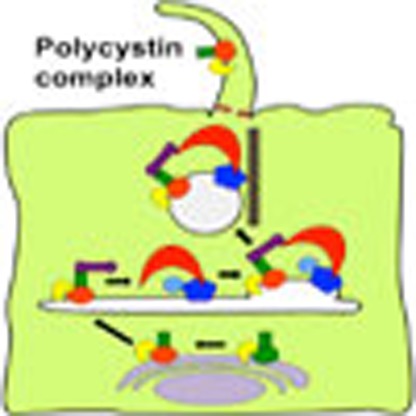

Primary cilia are microtubule-based non-motile projections on the apical surface of cells, which organize important signalling pathways mediated by specific receptors and channel proteins at the ciliary membrane in response to mechanical and chemical stimuli^1^. They are the key organelle for controlling correct tubular diameter, and defective ciliary function causes a variety of ciliopathies^1^, including autosomal dominant polycystic kidney disease (ADPKD)^2^,^3^. The ciliary membrane is separated from the plasma membrane by a periciliary diffusion barrier^4^, and ciliary membrane proteins must be transported to the cilia from their site of synthesis in the rough endoplasmic reticulum (ER) for proper ciliary function^5^. Experimental evidence favours a targeted delivery model, whereby ciliary membrane proteins are sorted in the Golgi and are targeted to the cilium by the vesicular pathway^5^. Numerous protein complexes have been implicated in polarized trafficking of post-Golgi vesicles to cilia, including the BBSome^5^ and intraflagellar transport complexes^6^. However, the mechanism by which transmembrane proteins are sorted for ciliary trafficking is poorly understood.

Polycystin-1 (PC1) and polycystin-2 (PC2), the two ADPKD gene products^7^,^8^, are large transmembrane proteins that co-localize to cilia^9^,^10^ where they play an important role in calcium-based signalling^2^,^11^. Loss of polycystin function in cilia is implicated in cyst formation^2^. PC1 is a 4,302-amino-acid atypical adhesion G-protein-coupled receptor (aGPCR) with 11-transmemrbane domains^12^,^13^. A fundamental property of PC1 is *cis*-autoproteolytic cleavage at a juxtamembrane G-protein-coupled receptor cleavage site (GPS)^14^,^15^,^16^. GPS cleavage of PC1 is developmentally regulated in the kidney, whereby PC1 is largely uncleaved in early embryonic kidneys but becomes extensively cleaved after birth^17^,^18^. Mice with a ‘knock-in’ missense amino-acid substitution that disrupts GPS cleavage (*Pkd1*^V/V^ mice) express non-cleavable PC1 (PC1^V^) and develop cystic kidney disease during the postnatal period^15^. As shown for other aGPCRs, cleavage results in a heterodimeric PC1 form, in which the N-terminal fragment (PC1_NTF_) remains stably and non-covalently associated with the transmembrane C-terminal fragment (PC1_CTF_)^14^,^19^. More recently, we have shown that GPS cleavage generates a complex pattern of endogenous PC1 forms but is not *per se* required for PC1 to move to the Golgi^18^. How GPS cleavage affects trafficking and function of PC1 or other aGPCRs *in vivo* remains unclear.

PC2 is a 968-amino-acid-long 6-transmembrane-spanning transient receptor potential channel family member that acts as a calcium release channel and is most abundantly distributed to the ER^20^,^21^. PC1 and PC2 form a receptor/channel complex by direct interaction via coiled-coil domains in their cytoplasmic C termini^22^,^23^,^24^. Several studies have reported that this interaction is required for surface membrane localization of the complex in certain, but not in all, cell types^25^,^26^,^27^. However, there is disagreement as to whether the interaction is necessary for ciliary localization, as each protein has its own ciliary targeting signal^25^,^28^,^29^. In some studies, PC2 was able to localize to cilia independently of PC1^28^,^30^, while other studies show that this requires PC1^2^,^25^,^31^,^32^. In addition, PC1 and PC2 may take different routes to reach the cilium. PC1 is described to traffic to cilia from the *trans*-Golgi network (TGN) via post-Golgi vesicles in an Arf4-dependent process^29^. However, PC2 was reported to move to the cilium directly from the *cis*-Golgi compartment without traversing the Golgi apparatus^30^.

In this report, we use a comprehensive approach to define the ciliary trafficking mechanism of endogenous polycystins in polarized ciliated renal epithelial cells. We demonstrate that PC1–PC2 interaction and GPS cleavage of PC1 are both required for the polycystin complex to reach the TGN and for subsequent ciliary targeting. We use yeast two-hybrid screening coupled with a candidate approach to identify a novel protein complex composed of Rabep1, GGA1 and Arl3, which is responsible for the sorting and targeting of the polycystin complex to the cilium. Our study provides novel insights into the ciliary trafficking mechanism of transmembrane proteins, with implications for ADPKD, the most common human ciliopathy.

## Results

### Polycystin complex is required for ciliary localization

**Figure 1:**
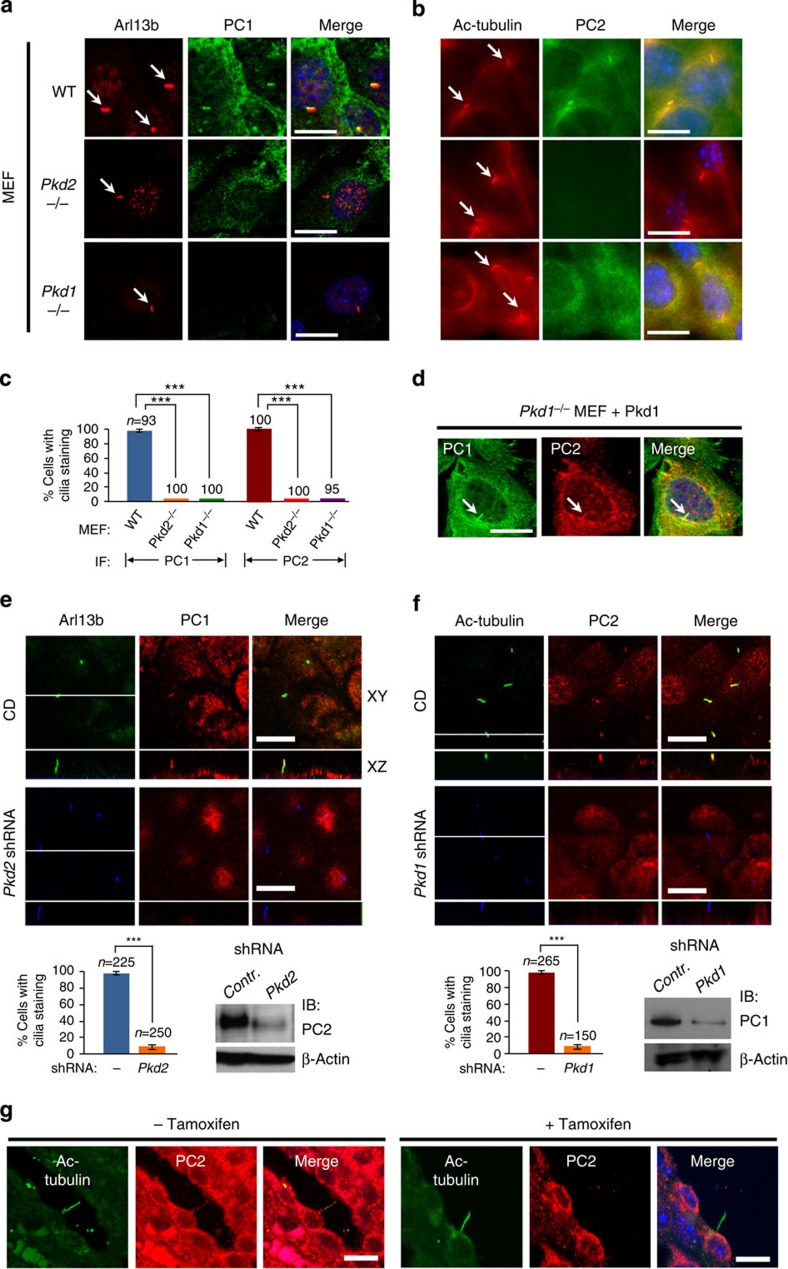
Native PC1 and PC2 regulate each other’s ciliary localization *in vivo*. (**a**,**b**) Representative immunofluorescence images of MEFs with indicated genotypes subjected to 24 h serum starvation. Cells were stained with anti-PC1 (E4, Supplementary Fig. 5) and analysed by confocal microscopy (**a**), or stained with anti-PC2 and analysed by epifluorescence microscopy (**b**). Arl13b or acetylated (Ac)-tubulin was used as a ciliary marker. Arrows indicate cilia. (**c**) The bar diagram shows the quantification of PC1 or PC2 ciliary localization in MEFs with various genotypes with numbers of cells analysed (*n*) indicated; ***P<0.001. (**d**) Exogenously expressed PC1 traffics to cilia (arrow) and rescues ciliary localization of PC2 in *Pkd1*^*−/−*^ MEFs. Exogenous expression of GFP served as a negative control (Supplementary Fig. 1). (**e**) Confocal images of CD cells (upper panel) and CD cells expressing *Pkd2* shRNA (lower panel). Cells were stained with antibodies against Arl13b (blue) and PC1(E4) (red). The bar diagram below shows the quantification of PC1 ciliary localization in CD and cells with *Pkd2* knockdown performed from four independent experiments with numbers of cells analysed (*n*) indicated; ***P<0.001. Western blot analysis shows that *Pkd2* shRNA reduced endogenous PC2 level by ~90%. (**f**) Confocal images of CD cells (upper panel) and cells expressing *Pkd1* shRNA, stained with antibodies as indicated. Four different *Pkd1* shRNAs had similar results. The bar diagram shows the quantification of PC2 ciliary localization between CD and cells with *Pkd1* knockdown from four independent experiments; ***P<0.001. Western blot analysis shows that *Pkd1* shRNA reduced endogenous PC1 level by ~90%. The white line in the XY scan in (**e**,**f**) indicates the path of the XZ scan. (**g**) Confocal images of PC2 ciliary localization in collecting duct at P28 in *Pkd1*^cko/cko^:tamoxifen-Cre mouse kidney^33^, in which *Pkd1* was not inactivated (left panel, normal tubule) or inactivated by tamoxifen injection on P10 (right panel, cystic tubule). PC2 was detected in 21 of 21 cilia (red) in normal kidneys. Cilia were marked by acetylated tubulin (green). Note that PC2 was not detectable in 18 of 18 cilia of cysts analysed. Scale bar, 10 μm.

We used several strategies to determine whether native PC1 and PC2 might regulate each other’s ciliary localization *in vivo*. We found that PC1 localizes to cilia in wild-type mouse embryonic fibroblasts (MEFs), but not in *Pkd2*^−/−^ MEFs (Fig. 1a,c). PC2 also localizes to cilia in wild-type MEFs, but not in *Pkd1*^−/−^ MEFs (Fig. 1b,c). Furthermore, exogenous mouse PC1 expressed in *Pkd1*^−/−^ MEF cells trafficked to the cilia and concomitantly restored ciliary localization of endogenous PC2 (Fig. 1d). The rescue of ciliary PC2 localization was not seen when GFP was expressed (Supplementary Fig. 1). We then used shRNA to knock down endogenous *Pkd2* expression in DBA-positive collecting duct (CD)-derived cells^15^, and we found that the ciliary localization of PC1 was abolished (Fig. 1e). Likewise, the ciliary localization of PC2 was not detectable when *Pkd1* expression was knocked down (Fig. 1f). The interdependence of PC1 and PC2 ciliary localization was confirmed in IMCD cells with stable expression of full-length epitope-tagged mouse PC1 (IMCD^PC1WT^, Supplementary Fig. 2a–e). In mice with a floxed *Pkd1* allele, PC2 was absent in the cilia of cystic kidney tubules after postnatal *Pkd1* inactivation^33^, while the protein was detected in the cilia of the normal tubular epithelial cells (Fig. 1g).

**Figure 2:**
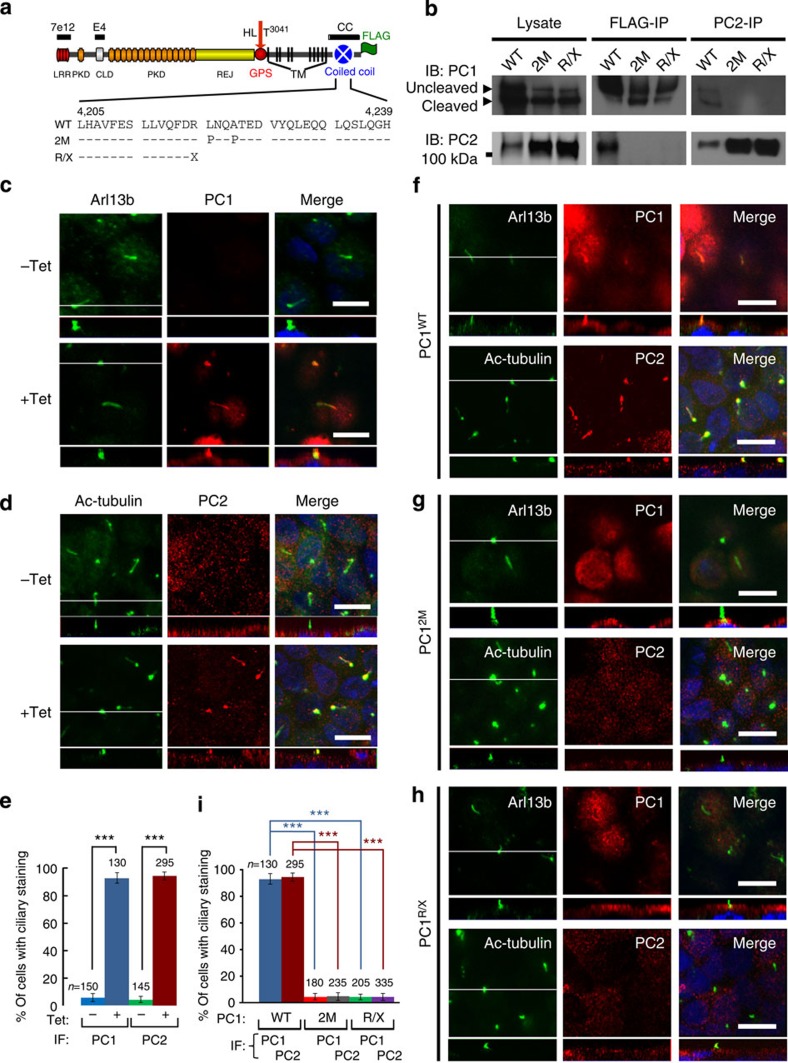
Direct interaction of PC1 and PC2 is required for ciliary localization. (**a**) A schematic diagram of the domain organization of mouse polycystin-1 with epitope position of antibodies and FLAG-tag indicated. LRR, leucine-rich repeat; CLD, C-type lectin-binding domain; PKD, polycystic kidney disease repeats; REJ, receptor for egg jelly module; GPS, G-protein-coupled receptor proteolytic site; TM, transmembrane domain. GPS cleavage occurs at HL^T^3041^. Mutations in the coiled-coil domain are indicated below the wild-type (WT) sequence. (**b**) Lysates of the induced MDCK cells were subject to reciprocal immunoprecipitation (IP) with the anti-FLAG or anti-PC2. The IP products were analysed by western blotting with antibodies against PC1 (7e12) or PC2 as indicated. (**c**,**d**) Confocal images of non-induced (−Tet) or induced (+Tet) MDCK^PC1WT^ cells, stained with antibodies as indicated. The white line in the XY scan indicates the path of the XZ scan. (**e**) Quantification of PC1 and PC2 ciliary localization for (**c**,**d**) from three independent experiments and presented as the mean±s.e.m.; ***P<0.001. The number of cells analysed is indicated. (**f**–**h**) Confocal images of induced MDCK cells expressing wild-type or mutant PC1 proteins as indicated, stained with antibodies as indicated. Note that the mutants do not localize to cilia nor induce ciliary localization of endogenous PC2. Scale bar, 10 μm. (**i**) Quantification of PC1 or PC2 ciliary localization in (**f**–**h**) from three independent experiments and presented as the mean±s.e.m.; ***P<0.001. The number of cells analysed is indicated.

We then tested whether direct interaction of PC1 and PC2 was required for ciliary localization of the polycystin complex. In MDCK cells with stable and inducible expression of epitope-tagged wild-type mouse PC1 (MDCK^PC1WT^, Table 1), we confirmed that recombinant PC1 formed a complex with endogenous PC2 (Fig. 2a,b). We found that, on induction of its expression, PC1 trafficked to cilia and concomitantly induced ER-resident endogenous PC2 to translocate to cilia as well (Fig. 2c–e). We disrupted the interaction of PC1 and PC2 by introducing amino-acid substitutions at two strategic positions (L4219P and A4222P) within the coiled-coil domain^22^,^24^ (PC1^2M^) (Fig. 2a,b). In contrast to wild-type PC1 (Fig. 2f), we found that PC1^2M^ did not traffic to the cilia and did not induce endogenous PC2 to translocate to the cilia (Fig. 2g,i). Similarly, PC1^R4218X^ (R/X) mutant^34^, which corresponds to an ADPKD patient-derived truncating mutant R4227X lacking the intact coiled-coil domain^22^,^27^, did not interact with endogenous PC2 (Fig. 2a,b) and failed to cause PC2 to translocate to cilia (Fig. 2h,i). Taken together, our results show that PC1 and PC2 must interact and form a complex for ciliary localization to occur in renal tubular epithelial cells.

### Polycystin complex is required to reach the Golgi apparatus

**Figure 3:**
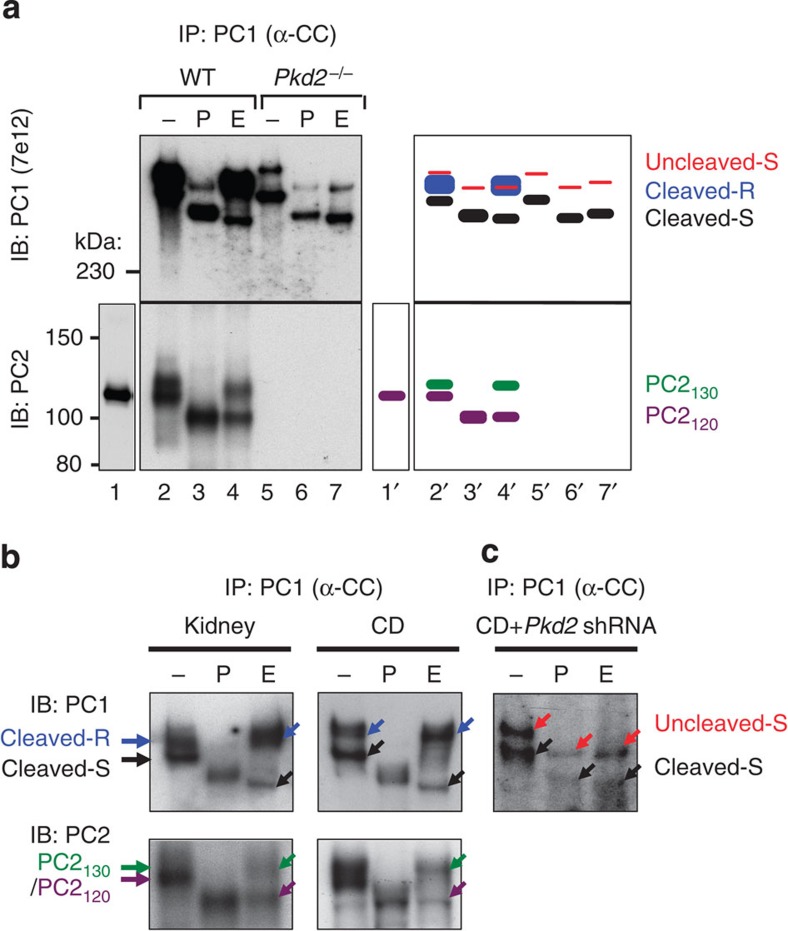
Polycystin complex formation is required to reach the Golgi apparatus. (**a**) *N*-glycosylation analysis of endogenous polycystin complex in WT and *Pkd2*^−/−^ MEFs. Polycystin complex was immunoprecipitated with anti-PC1 (α-CC), treated with PNGaseF (P) or EndoH (E), and analysed by western blot analysis with anti-PC1 (upper panel) or PC2 (lower panel). The schematic diagram at right provides an identification guide for various PC1 and PC2 forms, with colour code that is maintained throughout the figures. Note that PC2 is co-immunoprecipitated from WT MEFs as EndoH-resistant PC2_130_ and EndoH-sensitive PC2_120_; and cleaved PC1 cannot acquire EndoH resistance in *Pkd2*^−/−^ MEFs. (**b**) *N*-glycosylation analysis of endogenous polycystin complex in kidneys (left) and CD cells (right) as in (**a**). Note that EndoH-resistant PC2_130_ is detected in the polycystin complex in the kidney and CD lysates. (**c**) *N*-glycosylation analysis of polycystin complex in CD cells with *Pkd2* knockdown. Note that *Pkd2* knockdown prevented cleaved PC1 to acquire EndoH resistance. EndoH-sensitive uncleaved PC1 (~520 kDa) is visible as in *Pkd2*^−/−^ MEFs.

We next analysed the *N*-glycosylation pattern of the polycystin complex to investigate how it travels through the cell. EndoH cleaves high-mannose *N*-glycans attached in the ER, but not the *medial*/*trans*-Golgi-modified complex *N*-glycans^35^. EndoH resistance is thus indicative that glycoproteins have reached the *medial*/*trans*-Golgi compartment. Endogenous PC1 was immunoprecipitated with anti-CC (Fig. 2a) from MEFs, and the resulting precipitate was treated with EndoH followed by analysis with SDS–polyacrylamide gel electrophoresis (SDS–PAGE) and western blotting. In wild-type MEFs, PC1 was predominantly present as GPS-cleaved forms, which are recognized as the EndoH-resistant (‘Cleaved-R’) and -sensitive (‘Cleaved-S’) PC1_NTF_ bands (Fig. 3a, upper panel, lanes 2–4/2′-4′) as previously described^18^. Under these conditions, we observed very low levels of uncleaved PC1. In the whole-cell lysate, PC2 was detected as a single 120-kDa band (lower panel, lane 1/1′) as previously shown^20^,^21^. However, the fraction of PC2 that is co-immunoprecipitated with PC1 appeared as two distinct bands of similar intensity (lane 2/2′). Treatment of this PC2 fraction with EndoH revealed that the upper band (130-kDa, PC2_130_) was EndoH resistant, whereas the lower band (120-kDa, PC2_120_) was EndoH sensitive (lane 4/4′). A similar pattern was found in both kidney tissues and CD cells (Fig. 3b). These results identify a significant amount of EndoH-resistant pool of polycystin complex *in vivo*. In *Pkd2*^−/−^ MEFs, GPS-cleaved PC1 remained entirely EndoH-sensitive (Fig. 3a, upper panel, lanes 5–7), implying that PC1 cannot reach the *trans*-Golgi without PC2. Likewise, in CD cells with *Pkd2* knockdown, cleaved PC1 remains EndoH sensitive (Fig. 3c). Together, these data indicate that PC1 and PC2 form a complex in the ER and that direct interaction is required for the complex to reach the Golgi apparatus.

### Polycystin complex traffics to cilia through the Golgi

**Figure 4:**
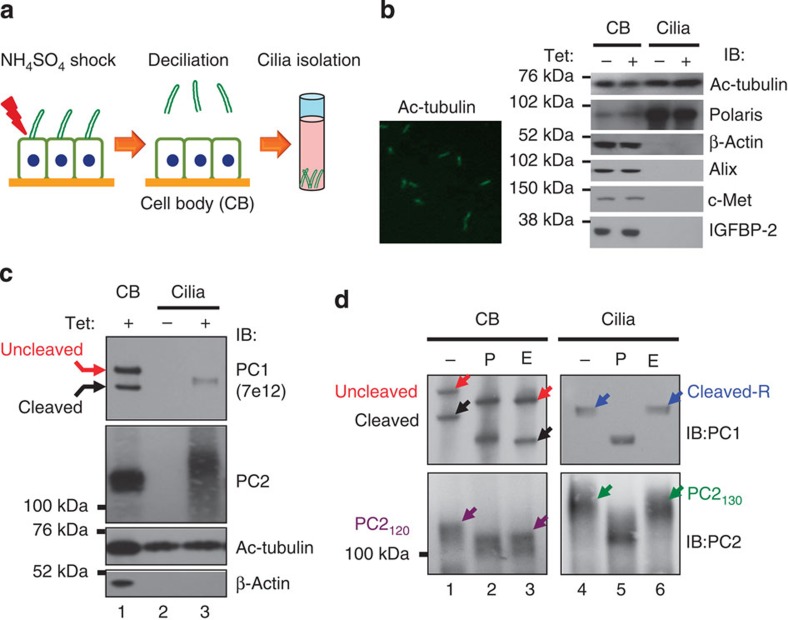
Polycystin complex traffics to cilia through the Golgi apparatus. (**a**) Schematic diagram of cilium isolation from MDCK monolayers. (**b**) Visualization of intact cilia in the cilium preparation by immunofluorescence with anti-Ac-tubulin (left). Right panel shows the validation of the cilium preparations by western blot analysis for marker proteins as indicated at right. The cilium preparation (Cilia) and the cell body (CB) without (‘−’) or with (‘+’) tetracycline induction were analysed. (**c**) Western blot analysis of cilia isolated from non-induced (‘−’) and induced (‘+’) MDCK^PC1WT^ cells with anti-PC1 or anti-PC2. (**d**) *N*-glycosylation analysis of ciliary PC1 and PC2 for the same sample as in (**c**). Parallel analysis of cell body (CB) served to identify cleavage status of ciliary polycystins. Note that only cleaved and EndoH-resistant PC1 and EndoH-resistant PC2_130_ forms are present in cilia.

To determine intracellular trafficking of ciliary PC1 and PC2, we isolated intact cilia from MDCK cells similarly as previously described^36^ (Fig. 4a) and analysed their *N*-glycosylation patterns. The preparation contained intact cilia as visualized by IF microscopy with a ciliary marker (Fig. 4b). Moreover, western blot analysis of this ciliary preparation detected acetylated tubulin and Polaris (known ciliary proteins), but not β-actin (an abundant cytoskeletal protein), Alix (an abundant component of exosome proteomes)^37^, c-Met (an apical plasma membrane protein)^38^ or IGFBP-2 (a secreted protein)^39^ (Fig. 4b). These results thus validated that the cilium preparation did not contain a detectable amount of contamination from intracellular and secreted proteins, exosomes or plasma membrane. We detected a single ~450-kDa PC1 band in the cilium preparation from induced MDCK^PC1WT^ cells (Fig. 4c, upper panel, lane 3), but not from non-induced cells (lane 2) or MDCK^pcDNA5^ control cells (data not shown). The ciliary PC1 migrated between the uncleaved PC1 and cleaved PC1 detected in the same cells stripped of cilia (cell body) (lane 1). Ciliary PC1 was resistant to EndoH (lane 3), but migrated at the expected molecular weight (MW) of cleaved PC1_NTF_ (~370 kDa)^14^ after removal of *N*-glycan by PNGaseF treatment (Fig. 4d, upper panel, lanes 2 and 5). This result identified the ciliary PC1 as the cleaved EndoH-resistent form. The uncleaved PC1 was absent in the cilia, but it was present at similar levels as cleaved PC1 in the cell body. These results indicate that only cleaved PC1 reaches the cilia and that it traffics through the Golgi. Remarkably, we detected a PC2 band of a MW of ~130 kDa in the cilium preparation from induced MDCK^PC1WT^ cells (Fig. 4c, lane 3), which migrated slower than the ~120-kDa PC2 band detected in the whole-cell body lysate (lane 1). The ciliary PC2 was also resistant to EndoH (Fig. 4d, lane 6) and migrated at the expected MW of PC2 after PNGaseF treatment (lane 5), as the PC2_130_ seen in endogenous polycystin complex from native tissues and cells (Fig. 3). Taken together, the presence of exclusively EndoH-resistant PC1 and PC2 in the cilia provides direct biochemical evidence that the polycystin complex traffics to cilia through the Golgi apparatus.

### Ciliary trafficking of polycystins requires GPS cleavage

**Figure 5:**
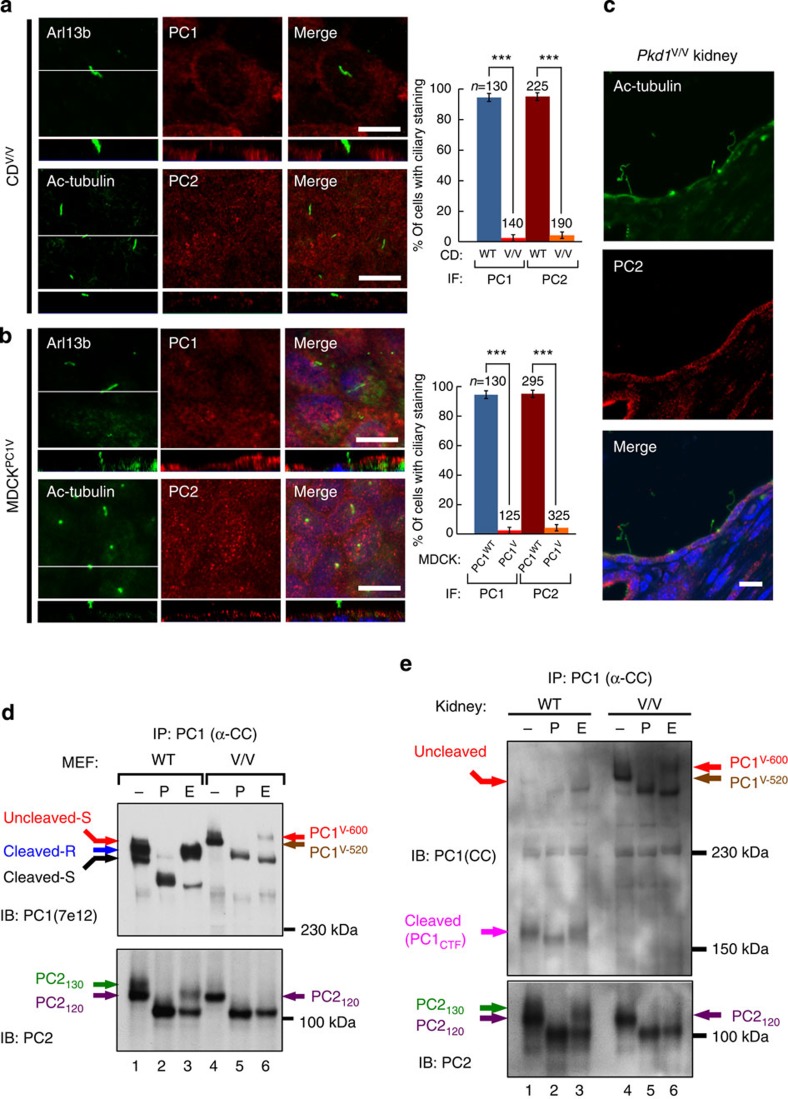
GPS cleavage is required for the ciliary trafficking of polycystin complex. (**a**) Confocal images of collecting duct cells isolated from *Pkd1*^V/V^ kidneys (CD^V/V^) stained with antibodies against PC1(E4) or PC2. Arl13b or Ac-tubulin was used as a ciliary marker. The graph compares the percentage of cells with positive PC1 or PC2 in the wild-type CD (as shown in Fig. 1e or f) and CD^V/V^ cells, from three independent experiments and presented as the mean±s.e.m.; ***P<0.001. The number of cells analysed (*n*) is indicated. (**b**) Confocal images of induced MDCK^PC1V^ cells show the absence of ciliary PC1^V^ and endogenous PC2. The graph compares the percentage of cells with positive PC1 or PC2 ciliary staining in MDCK^PC1WT^ (as shown in Fig. 2f) and MDCK^PC1V^ cells from three independent experiments, presented as the mean±s.e.m.; ***P<0.001. The number of cells analysed (*n*) is indicated. The white line in the XY scan in (**a**,**b**) indicates the path of the XZ scan. Scale bar, 10 μm. (**c**) Confocal images of cystic collecting ducts of the *Pkd1*^V/V^ kidney stained with anti-PC2. Note that PC2 was not detectable in 17 of 17 cilia analysed. (**d**) *N*-glycosylation pattern of polycystin complex in *Pkd1*^V/V^ MEFs versus wild-type MEFs. Polycystin complex was immunoprecipitated and analysed as in Fig. 3, with PC1 and PC2 bands indicated with colour code. Note that the co-precipitated PC2 from *Pkd1*^V/V^ MEFs (lanes 4–6) was entirely EndoH sensitive, lacking EndoH-resistant PC2_130_ seen in wild-type MEFs (lanes 1–3). (**e**) *N*-glycosylation pattern of polycystin complex in *Pkd1*^V/V^ kidney versus wild-type kidney. Polycystin complex was immunoprecipitated and analysed as in (**d**), expect that an anti-CC antibody was used to detect PC1. Note the presence of cleaved PC1_CTF_ in wild-type kidney (lanes 1–3) but not in *Pkd1*^V/V^ kidneys (lanes 4–6).

The absence of uncleaved PC1 in the cilia suggests that GPS cleavage is required for ciliary trafficking of the polycystin complex. To test this we asked whether the non-cleavable PC1^V^ could be found in cilia. In fact we were unable to detect either ciliary PC1^V^ or PC2 in the cilia of CD cells derived from *Pkd1*^V/V^ kidneys (CD^V/V^) (Fig. 5a). In addition, recombinant PC1^V^ overexpressed in MDCK (MDCK^PC1V^, Table 1) was unable to reach the cilia and did not induce endogenous PC2 to translocate to cilia (Fig. 5b). We obtained similar results in IMCD^PC1V^ cells (Supplementary Fig. 2f). Consistent with this result, we were unable to detect either PC1^V^ or PC2 in the cilium preparation from induced MDCK^PC1V^ cells by western blot analysis (data not shown). Importantly, PC2 was not detectable in the cilia of cystic tubules of the *Pkd1*^V/V^ kidneys (Fig. 5c).

To investigate the basis of the observed requirement of GPS cleavage, we analysed the *N*-glycosylation pattern of the polycystin complex in *Pkd1*^V/V^ MEFs versus wild-type MEFs (Fig. 5d). The non-cleavable PC1^V^ was immunoprecipitated from *Pkd1*^V/V^ MEFs as an ~600-kDa EndoH-resistant band (PC1^V-600^) and an ~520-kDa EndoH-sensitive band (PC1^V-520^) (upper panel, lanes 4–6). We found that PC2 was still co-precipitated by PC1^V^, but the co-precipitated PC2 appeared only as an ~120-kDa band and was completely sensitive to EndoH (that is, PC2_120_, lanes 4–6), lacking the EndoH-resistant PC2_130_ seen in WT MEFs (lanes 1–3). The same *N*-glycosylation pattern was found for the polycystin complex in *Pkd1*^V/V^ kidney tissues (Fig. 5e). These results imply that only the EndoH-sensitive PC1^V-520^, but not the EndoH-resistant PC1^V-600^, is associated with PC2 in the *Pkd1*^V/V^ samples. Our data collectively show that GPS cleavage is required for the ciliary localization of the polycystin complex by enabling its trafficking to the *trans*-Golgi compartment. In the absence of cleavage, the PC1^V^/PC2 complex cannot reach the *trans*-Golgi.

### Rabep1 binds PC1 C-terminus for ciliary trafficking

**Figure 6:**
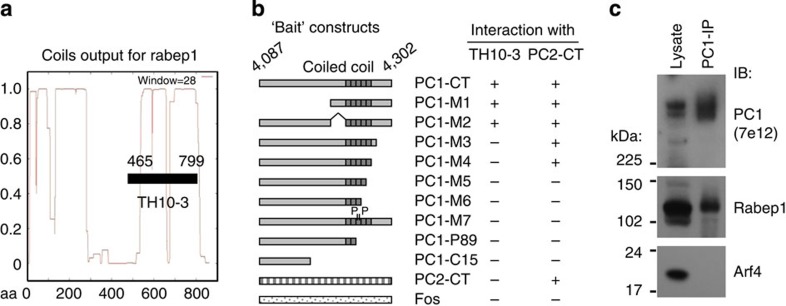
Interaction of PC1 cytoplasmic C-terminal tail with Rabep1. (**a**) Yeast two-hybrid screening of a rat brain cDNA library using human PC1 215-amino-acid C-terminal tail as bait (PC1-CT) identified a clone (TH10-3) corresponding to amino acids 465 to 799 of Rabep1 with extensive coiled-coils^69^. (**b**) Mapping of Rabep1 interaction region by yeast two-hybrid assay using a series of PC1-CT deletion constructs^22^. The coiled-coil domain is indicated. PC1-M7 contains proline at *a* and *d* positions of the heptad, disrupting α-helical structure of the coiled-coil domain. PC1-P89 contains a germline mutation R4227X^22^,^27^. PC1-C15 contains a somatic mutation found in a renal cyst resulting in reading-frame shift^22^,^70^. Positive interaction was scored by both growth on triple selective medium and positive β-gal activity. PC2 C-terminal tail (PC2-CT) or Fos served as negative controls. Interactions of the PC1 constructs with PC2-CT are shown as a comparison. (**c**) Co-immunoprecipitation of endogenous PC1 with Rabep1, but not with Arf4 in CD cells.

To identify the molecules that mediate polycystin complex ciliary trafficking, we performed a yeast two-hybrid screen for PC1-interacting proteins using the entire 215-amino-acid C-terminal tail as bait. Thirteen unique cDNA clones were identified that specifically interact with the PC1 C-terminus. One of these clones corresponded to amino acids 465 to 799 of Rabep1 (Fig. 6a), an effector of multiple Rab GTPases involved in various steps of intracellular vesicular trafficking^40^. This protein was chosen for further analysis. We used mutational analysis to map the Rabep1 interaction region (Fig. 6b) and showed that the C-terminal 89 amino acids containing the intact coiled-coil domain of PC1 (PC1-M1/M2) was sufficient for interaction with Rabep1. The binding sites for Rabep1 and PC2 overlap but are not identical, as the C-terminal 41 amino acids of PC1 (PC1-M3) was required for Rabep1 interaction, but not for PC2.

**Figure 7:**
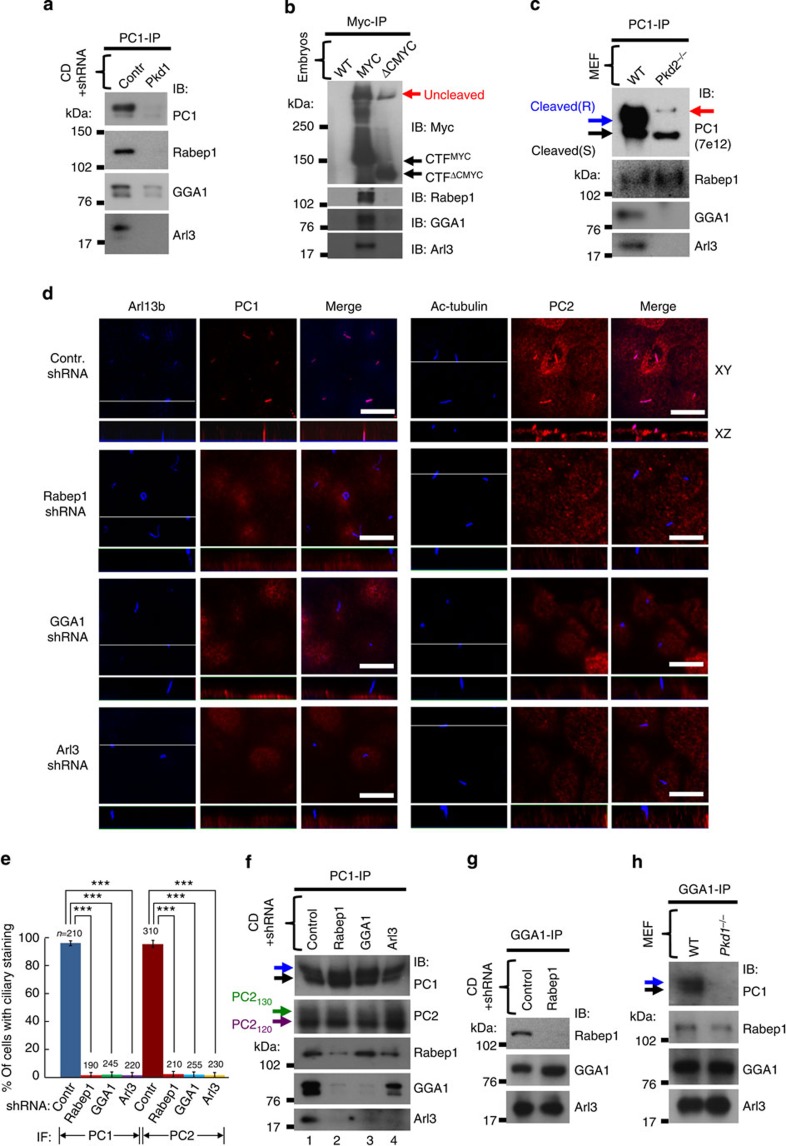
Polycystin complex containing cleaved PC1 traffics to cilia through a Rabep1/GGA1/Arl3-dependent mechanism. (**a**) Co-immunoprecipitation of endogenous PC1 with Rabep1, GGA1 and Arl3 from CD cells. CD cells with shRNA-mediated *Pkd1* knockdown were used as a negative control. The larger areas of the blots are shown in Supplementary Fig. 3d. (**b**) Co-immunoprecipitation of C-terminal Myc-tagged PC1 with Rabep1, GGA1 and Arl3 from *Pkd1*^MYC/MYC^ embryo lysates (MYC). *Pkd1*^ΔCMYC/ΔCMYC^ knockout embryos that expresses truncated PC1 lacking the C-terminal 257 amino acids (ΔCMYC) and untagged wild-type (WT) embryos were used as negative controls. (**c**) Wild-type (WT) or *Pkd2*^−/−^ MEF cell lysates were immunoprecipitated with anti-PC1 (α-CC), and the IP products were analysed with the antibodies as indicated. (**d**) Knockdown of Rabep1, GGA1 or Arl3 in CD cells abolishes ciliary localization of endogenous PC1 (left panels) and PC2 (right panels). The white line in the XY scan indicates the path of the XZ scan. For knockdown of each target protein, three shRNAs for Rabep1, four shRNAs for GGA1 and two shRNAs for Arl3 were used, and each lentiviral shRNAs similarly inhibit the expression of the target proteins by ~90% (Supplementary Fig. 3a–c). Scale bar, 10 μm. (**e**) Quantification of PC1 and PC2 ciliary localization in CD cells expressing various shRNAs in (**d**) was performed from four independent experiments and presented as the mean±s.e.m.; ***P<0.001. (**f**) Endogenous PC1 were immunoprecipitated with anti-PC1 (α-CC) from CD cells expressing the shRNA as indicated and analysed with various antibodies as indicated. Note that acquisitions of EndoH resistance of PC1 and PC2 were not affected by any of the shRNAs. The larger areas of the blots are shown in Supplementary Fig. 3e. (**g**) Co-immunoprecipitation of native GGA1 and Arl3 in CD cells with Rabep1 knockdown. The specificity of the GGA1–Arl3 interaction was validated using CD cells with GGA1 knockdown (Supplementary Fig. 4d). (**h**) Co-immunoprecipitation of GGA1 with Rabep1 and Arl3 in both wild-type (WT) and *Pkd1*^−/−^ MEFs.

We confirmed that the PC1–Rabep1 interaction occurs *in vivo* by demonstrating co-immunoprecipitation from CD cells (Fig. 6c). We were unable to co-immunoprecipitate these two proteins from CD cells with *Pkd1* knockdown (Fig. 7a) nor from *Pkd1*^ΔCMYC/ΔCMYC^ knockout embryos expressing Myc-tagged truncated PC1 (ref. 41) (Fig. 7b). We found that the PC1–Rabep1 interaction occurred in *Pkd2*-null MEFs (Fig. 7c). This suggests that PC1 binds Rabep1 in a pre-Golgi compartment, as PC1 does not exit the ER in the absence of PC2 (Fig. 3a).

Next we tested whether Rabep1 was required for ciliary localization of the polycystin complex. We used shRNA to knock down Rabep1 in CD cells, and this abolished ciliary localization of PC1 and PC2 (Fig. 7d,e). Rabep1 knockdown in the CD cells, however, did not affect formation of the polycystin complex nor its acquisition of EndoH resistance (Fig. 7f, lane 2), indicating that Rabep1 is not required for the polycystin complex to reach the Golgi. Collectively, these results show that Rabep1 binds to the distal portion of PC1_CTF_’s C-terminal tail and suggest that Rabep1 likely plays a critical role for ciliary targeting of the polycystin complex at the TGN by recruiting ciliary targeting machinery.

### Rabep1 recruits polycystin complex to GGA1 and Arl3 at TGN

Rabep1 has been shown to interact with GGA1 (refs 42, 43, 44) (Golgi-localized, gamma adaptin ear-containing, ARF-binding) in the context of fusion of TGN-derived vesicles with endosomes^45^. GGA1 is an effector of Arf GTPases at the TGN and mediates protein sorting and vesicle budding from the TGN^43^,^46^. We therefore hypothesized that polycystin complex-associated Rabep1 might bind GGA1, which in turn might mediate the ciliary sorting of the complex. Indeed, we found that PC1 can co-immunoprecipitate GGA1, along with PC2 and Rabep1, from CD cell lysates (Fig. 7a,f) and *Pkd1*^MYC/MYC^ embryos (Fig. 7b). GGA1 knockdown abolished the ciliary localization of both PC1 and PC2 in CD cells (Fig. 7d,e), without affecting the acquisition of EndoH resistance of the polycystin complex (Fig. 7f, lane 3). PC1–GGA1 interaction was abolished by Rabep1 knockdown (lane 2), whereas PC1–Rabep1 interaction still occurred when GGA1 was knocked down (lane 3), indicating that Rabep1 bridges an interaction between PC1 and GGA1. Our data provide evidence that GGA1 is recruited to the polycystin complex by Rabep1 and plays a critical role for subsequent ciliary targeting from the TGN.

GGA proteins are recruited to the TGN membrane via their direct interaction with specific Arf members^43^,^46^. As GGA1 affected by the known Arfs such as Arf1 (ref. 47) or Arf3 (ref. 42) has not been shown to be involved in ciliary trafficking, we reasoned that additional small GTPases would likely be required to confer specificity of polycystin complex ciliary targeting. A previous study using a small CD16.7-PC1 chimeric construct encoding the last 112 amino acids of the PC1 cytoplasmic C-terminus^29^ has implicated Arf4 (ref. 48) in the ciliary trafficking of PC1. We have asked whether Arf4 may cooperate with GGA1 at the polycystin complex. However, we were unable to detect Arf4 in the PC1 complex from the CD cell lysates (Fig. 6c). Arl3, a closely related member of the Arf family, localizes to the Golgi membrane and other microtubule-related structures including cilia^49^,^50^. Remarkably, Arl3 inactivation in mice results in cystic kidney disease^51^, reminiscent to what is found in *Pkd1*^V/V^ mice lacking PC1 GPS cleavage^15^. We thus hypothesized that Arl3 binds GGA1 on polycystin complex to regulate its ciliary targeting. Indeed, PC1 co-immunoprecipitated both Arl3 and GGA1, along with Rabep1 and PC2 (Fig. 7a,b). Importantly, Arl3 knockdown in CD cells abolished the ciliary localization of PC1 and PC2 (Fig. 7d,e), indicating a critical role of Arl3 for the ciliary targeting of the polycystin complex.

We then analysed mutual requirements of Arl3, GGA1 and Rabep1 for PC1 binding. Rabep1 knockdown disrupted the interaction of PC1 with both GGA1 and Arl3 (Fig. 7f, lane 2), whereas the PC1–Rabep1 interaction still occurred when GGA1 or Arl3 was knocked down (lane 3 or 4). Arl3 knockdown, however, did not disrupt the interaction of PC1 with Rabep1 or GGA1 (lane 4). These results raised the possibility that Rabep1 may recruit a GGA1/Arl3 complex to the polycystin complex by interacting with GGA1. In support of this notion, we found that endogenous GGA1 and Arl3 can be co-immunoprecipitated from CD cells depleted of Rabep1 (Fig. 7g) or in *Pkd1*^−/−^ MEFs (Fig. 7h). Furthermore, when retained in a pre-Golgi compartment in *Pkd2*^−/−^ MEFs, PC1–Rabep1 complex did not bind GGA1 or Arl3 (Fig. 7c). Collectively, our data support a model in which Rabep1 recruits the polycystin complex to GGA1/Arl3 at TGN for ciliary trafficking.

## Discussion

To study ciliary membrane protein trafficking, we focused on two large transmembrane proteins, PC1 and PC2, the products of the genes mutated in human ADPKD. Our study shows that polycystin trafficking to the cilium occurs in a two-step process. First, PC1 and PC2 must interact and form a complex to traffic from the ER to the Golgi. In addition, PC1 must be cleaved at GPS for this to occur. Rabep1 binds the polycystin complex at PC1 C-terminal tail and rides on the complex to the Golgi, but is not itself required for this step in trafficking. Second, once the polycystin complex-bound Rabep1 reaches the TGN, it binds GGA1 that is associated with Arl3 for subsequent targeting to the cilium.

Our finding that a direct PC1–PC2 interaction is required for ciliary trafficking is consistent with some^2^,^25^, but differs from other published studies^28^,^30^. Some of the discrepant findings may be due to the use of overexpression systems by several authors, while we have focused on analysing trafficking of endogenous proteins. It has been previously proposed that PC2 is continuously released from the ER to the Golgi in a COPII-dependent manner but immediately returned via an ER retention/retrieval signal in the C-terminus^20^,^30^,^52^. A more recent study reported that a small portion of PC2 escapes retrograde transport and traffics to the cilium directly from the *cis*-Golgi compartment without traversing the Golgi apparatus^30^. This was inferred in part from the complete EndoH sensitivity of PC2 at the whole-cell lysate level. We used co-immunoprecipitation to isolate the fraction of PC2 that is complexed with PC1 from native tissue and cell lysates, and found that a significant portion was EndoH resistant (PC2_130_). PC2_130_ is present in a rather small fraction of the total cellular PC2 and has not been recognized previously when analysed at whole-cell lysate levels, probably because it is obscured by the much more abundant EndoH-sensitive ER-resident PC2_120_ that migrates at a very close position on western blots. Our co-immunoprecipitation strategy simultaneously enriches the native polycystin complex and removes the predominant interfering EndoH-sensitive PC2_120_ form that is not complexed with PC1, thereby allowing the visualization of PC2_130_ of the polycystin complex. Our finding of exclusive EndoH resistance of both PC1 and PC2 in isolated cilia provided direct biochemical evidence that the polycystin complex traffics to cilia in fact through the Golgi apparatus. The ciliary polycystins may represent some of the EndoH-resistant polycystin complex population detected in native kidney tissues and cells. However, given the relatively small volume occupied by the cilium, most of this polycystin population probably resides on non-ciliary compartments, such as the Golgi or post-Golgi vesicles, or at the plasma membrane^18^. Further studies are required to resolve this issue.

PC1 is generally far less abundant than PC2 in kidneys and is the central determinant of cyst formation in polycystic kidney and liver diseases^53^,^54^. We find that ectopic expression of PC1 can induce the ER-resident endogenous PC2 to translocate to the cilium. These data support the idea that PC1 is the rate-limiting factor for ciliary trafficking of the polycystin complex in renal epithelial cells. We propose that the PC1–PC2 interaction is required to counteract and overcome the retrograde transport of the polycystin complex to the ER by masking the ER retention/retrieval signals or alternatively by allowing the complex to pass quality-control checkpoints in the ER as found for many GPCRs^55^. This allows the complex to efficiently reach the TGN for subsequent ciliary targeting by a Rabep1/GGA1/Arl3-dependent process.

GPS cleavage of PC1 is also required for the polycystin complex to move to the TGN. Nonetheless, the non-cleavable PC1^V^ can still move to the Golgi apparatus as evidenced by its ability of acquiring EndoH resistance (PC1^V-600^, Fig. 5d,e). One possible explanation is that PC1^V^ cannot remain associated with PC2 at the Golgi apparatus and consequently traffics to a non-ciliary location yet to be determined. Cleavage may promote the trafficking of the polycystin complex to the Golgi apparatus by increasing PC1/PC2 binding affinity, thereby inhibiting PC2 dissociation. Alternatively, cleavage could increase the ER-to-Golgi transition rate of the polycystin complex, thereby outpacing PC2 dissociation. GPS cleavage could be significant for regulating polycystin trafficking in the kidney in a development-specific manner. In early embryonic stages, PC1 exists largely in its uncleaved form, and therefore it might mainly traffic, perhaps apart from PC2, to a non-ciliary location such as at the cell–cell junction where it could regulate convergent extension and elongation of developing renal tubules^17^,^56^. After birth, however, PC1 is mostly cleaved^15^,^17^,^18^ and probably traffics to the cilia in the form of the polycystin complex to control proper tubular diameter^56^.

Previous efforts have focused on the identification of the ciliary-targeting motifs and binding proteins that may mediate ciliary trafficking^57^. Both PC1 and PC2 have been reported to contain ciliary-targeting signals. For PC1 the sequence is at its extreme C-terminus^29^, while for PC2 the ciliary targeting sequence is within the first 15 amino acids at the N-terminus^28^. Our findings indicate that the ciliary-targeting signal in each polycystin is not sufficient for ciliary trafficking in renal epithelial cells. We have identified a novel ciliary sorting module that is composed of Rabep1, GGA1 and Arl3, which is recruited to the polycystin complex by PC1, and is required for the ciliary targeting of the complex. Rabep1-PC1 binding requires the intact coiled-coil domain of PC1, which is outside of the previously reported ciliary targeting sequence^29^. The same study using a small CD16.7-PC1 chimeric construct encoding the last 112 amino acids of the PC1 cytoplasmic C-terminus^29^ has also implicated Arf4 in the ciliary trafficking of PC1. However, we were unable to detect Arf4 binding to full-length endogenous PC1 in CD cells, which makes it unlikely that Arf4 plays an important role in targeting of the polycystin complex.

GGA1 is a member of the monomeric GGA family that is known to select proteins at the TGN into clathrin- and GGA-coated carriers, which are then transported to endosomes. GGA1 exerts its function by the different domains that interact with various other proteins^44^. The GAT domain binds activated Arf-family GTP-binding proteins and thereby recruits GGA1 to the TGN. The hinge domain then binds clathrin for vesicle budding, while the GAE domain binds accessory factors that can regulate the function of GGA-coated carriers. Arl3 plays a critical role in ciliary function and is involved in intraflagellar transport in *C. elegans*^58^. In mice, *Arl3* inactivation results in various ciliopathy-related phenotypes including cystic kidney disease and retinal degeneration^51^. In addition to the cilium, Arl3 also has been shown to localize to the Golgi, but the function of this Golgi-associated pool is unknown^50^. We show for the first time that there is a distinct pool of Arl3 that is bound to GGA1. This GGA1/Arl3 module likely binds cargo and other components necessary for clathrin binding, forming vesicle carriers destined for the cilium. As Arl3 is known to have microtubule-binding activity^50^, one possibility is that Arl3 may direct the cargo-bearing vesicles to cytoplasmic microtubules for dynein-driven transport to the cilium. The dynein-dependent system has been reported to translocate rhodopsin-bearing vesicles along microtubules towards the cilium in polarized epithelia^59^.

**Figure 8:**
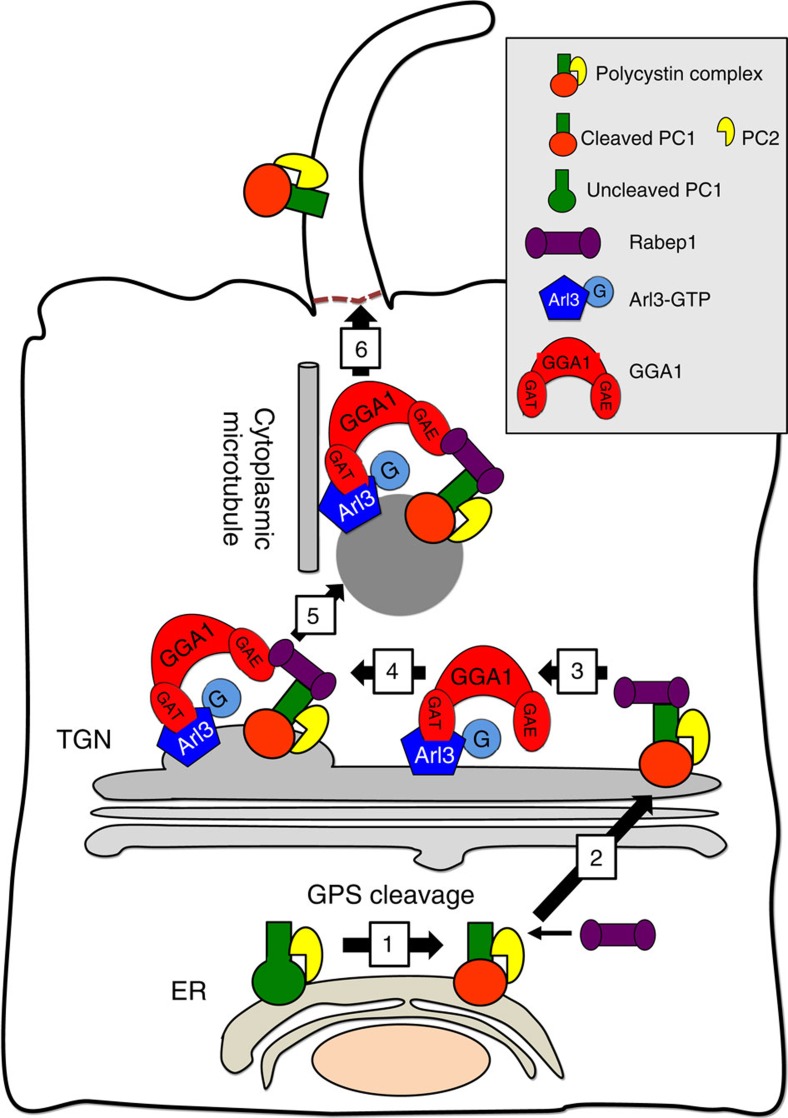
Model for ciliary trafficking of the polycystin complex. PC1 is cleaved at GPS and forms a complex with PC2 in the ER (1). Rabep1 binds PC1 cytoplasmic C-terminal tail at a pre-Golgi compartment (2), and this complex traffics to the *trans*-Golgi network (TGN). At the TGN, Rabep1 couples the polycystin complex to a GGA1/Arl3 module (3), which is formed by Arl3-GTP binding to GGA1 via the GAT domain. GGA1 then assembles the clathrin coat (4) to form the vesicle carrier (5). The resulting polycystin complex-bearing vesicle traffics along the cytoplasmic microtubules to the base of cilia (6) and enters the cilium by an unknown mechanism.

A central region of Rabep1 has been shown to interact with the GAE domain of GGA1 (ref. 45). The Rabep1–GGA1 interaction is bipartite, as the C-terminal coiled-coil region of Rabep1 also binds the GAT domain of GGA1. This bivalent interaction is thought to mediate fusion of post-Golgi GGA1-coated vesicles to Rabep1-bearing endosomes. PC1 binds the C-terminal coiled-coil region of Rabep1 that usually binds to GAT, thus likely leaving its central region accessible for interacting with the GAE domain of GGA1/Arl3. Our current model is that polycystin complex-bound Rabep1 serves as an accessory protein for GGA1 via its GAE domain, thereby coupling the polycystin complex to the GGA1/Arl3 module (Fig. 8). It remains to be determined whether this module may be involved in the later stages of ciliary trafficking as recently described for Rabep1 (ref. 60) and Arl3 (refs 61, 62). Further studies are also required to investigate how the Rabep1/GGA1/Arl3 complex is related to the previously identified trafficking complexes including the exocyst^63^ and BBSome^64^.

Our model has several important implications. First, this general mechanism could conceivably be used to transport the polycystin complex to other cellular locations. For example, the Rabep1–GGA1–polycystin complex could bind other members of the ARF family. This may result in trafficking to non-ciliary locations such as the plasma membrane where polycystins have also been detected^25^,^26^,^27^. Second, as the interactions between Rabep1, GGA1 and Arl3 can occur independently of PC1, Rabep1 may bind and couple other ciliary membrane proteins to the GGA1/Arl3 module for ciliary targeting. Third, as the interaction between GGA1 and Arl3 can occur independently Rabep1, one can imagine that accessory proteins other than Rabep1 may bind and couple yet other ciliary membrane proteins to GGA1/Arl3 via the GAE domain for ciliary targeting. In summary, this work provides novel insights into the mechanism of the ciliary trafficking of membrane proteins.

## Methods

### Cell lines

CD cells were affinity purified from mouse postnatal kidneys by using DBA-conjugated Dynabeads (Invitrogen). The kidneys were minced into small pieces and digested in MEM/F12 containing 0.2% collagenase, 0.2% hyaluronidase and 0.001% DNase I at 37 °C for 2 h with gentle agitation. The digested tissue was incubated with Dynabeads at 4 °C for 30 min. The cells bound to the beads were suspended in culture medium for propagation^15^. Tetracycline-inducible and stable MDCK and IMCD cells were generated using Flp-In System (Invitrogen)^65^,^66^ by transfecting pcDNA5-based wild-type or mutant *Pkd1* expression plasmids according to the manufacturer’s protocol. MDCK cell lines were cultured in DMEM growth media with 100 μg ml^−1^ hygromycin B and 5 μg ml^−1^ blasticidin. Stable IMCD cell lines were cultured in DMEM/F12 growth media with 50 μg ml^−1^ hygromycin B.

### Cilia preparation

MDCK cells were cultured on Transwell filter for ~10 days for cilium growth using DMEM with 10% FBS at the outside membrane and DMEM without FBS at the inside membrane of transwell plate. The cells were washed with PBS and treated with 30 mM ammonium sulfate for 3 h to shed cilia into the medium^36^. Intact shed cilia were collected from the medium by sequential centrifugation (at 2,000 *g* for 30 min, 10,000 *g* for 30 min and at 16,000 *g* for 30 min). After centrifugation, the cilia pellet was resuspended in PBS for immunofluorescence or western blot analysis.

### Immunofluorescence

Cultured cells were fixed in 4% formaldehyde for 15 min and then permeabilized by 0.1% Triton X-100 for 5 min, and incubated with primary antibodies in blocking buffer (5% FBS/ 0.05% Triton X-100 in PBS) for overnight. Cells were then incubated with fluorescence-conjugated secondary antibodies (Invitrogen, CA) for 1 h and mounted by VectaShield Mounting Medium (Vector Laboratories, CA). Confocal microscope LSM 510 (Carl Zeiss Microscopy) was used to acquire images. For paraffin-embedded kidney sections, sections were first deparaffinized by xylene for 10 min and rehydrated using a graded series of alcohol solutions, followed by antigen retrieval using Target Retrieval Solution, Citrate Buffer pH 6 (Dako, CA), and then followed the procedure as previously described^15^. The primary antibodies used for immunofluorescence included mouse anti-acetylated tubulin from Sigma-Aldrich (T7451, 1:4,000) and rabbit anti-Arl13b from Proteintech (17711-1-AP, 1:200) for cilia markers and rabbit anti-PC2 (ref. 34) (1:200).

### Immunoprecipitation and immunoblot analysis

Immunoprecipitation (IP) studies of the endogenous and recombinant PC1 were accomplished on mouse tissue samples and cells (MEFs, CD cells, MDCK) homogenized in lysis buffer (20 mM sodium phosphate, pH 7.2/150 mM NaCl/1 mM EDTA/10% (vol/vol) glycerol/1% Triton X-100/protease inhibitors)^15^. In brief, the homogenate was incubated with the primary antibody or antibody-conjugated bead for overnight, followed by incubation with G Sepharose 4 Fast Flow (GE Healthcare) or goat anti-chicken IgY agarose beads (PrecipHen, Aves) for 3 h. Bead pellets were washed with lysis buffer three times and eluded with SDS sample buffer. The primary antibodies and antibody-conjugated bead used for IP included mouse anti-FLAG M2 affinity gel from Sigma-Aldrich (A2220), rabbit anti-FLAG from Cell Signaling Technology (#2368, 1:50), chicken anti-PC1 (α-CC, 1-2 μg ml^−1^)^15^, rabbit anti-Myc from Cell Signaling Technology (#2278, 1:200) and rabbit anti-GGA1 from Thermo Scientific (PA5-12130, 1-2 μg ml^−1^). The protein samples were loaded on 3–8% Tris-Acetate SDS–polyacrylamide precast gels or 4–12% Tris-Glycine SDS–polyacrylamide precast gels (Invitrogen) and transferred to PVDF membrane (Bio-Rad). The membranes were incubated with primary antibodies for overnight and then washed with TBS-Tween 20 buffer. An HRP-conjugated secondary antibody from GE Healthcare (NA934V; 1:10,000) was incubated for 1 h, and then ECL Prime (GE Healthcare) was used for detection on a Kodak film. The membranes were then stripped using Restore Western blot buffer (Pierce, VWR) and reprobed. The primary antibodies used for immunoblotting included mouse anti-PC1 (7e12) from Santa Cruz Biotechnology (sc-130554, 1:1,000), mouse anti-β-actin from Sigma-Aldrich (A5316, 1:10,000), rabbit anti-Polaris from Thermo Scientific (PA5-18467, 1:1,000), mouse anti-Alix from Cell Signaling Technology (#2171, 1:1,000), mouse anti-Rabep1 from BD Bioscience (610676, 1:1,000), rabbit anti-GGA1 from Abcam (ab38454, 1:500), rabbit anti-c-MET from Cell Signaling Technology (#8198, 1:1,000), rabbit anti-IGFBP-2 (11065-3-AP, 1:1,000), rabbit anti-Arf4 (11673-1-AP, 1:1,000) and rabbit anti-Arl3 (10961-1-AP, 1:500) from Proteintech. The E4 monoclonal antibody against PC1 was generated from mice immunized with recombinant 130-amino-acid long polypeptide corresponding to residues 406–535 (C-lectin binding domain) of human PC1 protein. The specificity is shown in Supplementary Fig. 5.

### *N*-glycosylation analysis

Immunoprecipitation products or lysates were denatured using glycoprotein denature buffer (New England Biolabs) for 1 min at 95 °C and then quickly chilled on ice. The denatured glycoprotein was incubated with PNGaseF or EndoH (New England Biolabs) for 1 h at 37 °C.

### shRNA sequences and lentiviral infection

pGIPZ Lentiviral shRNAs (Thermo Scientific) were used to inhibit the expression of target genes. For PC1 knockdown, we used three pGIPZ Lentiviral shRNAs containing following antisense sequences: *Pkd1* antisense #1; 5′-AAGCCAATGAGGTCACCAG-3′, *Pkd1* antisense #2; 5′-AACGCAGCAGTAATCTGCT-3′ and *Pkd1* antisense #3; 5′-TTCTCTCCAGGAACACTGG-3′. We have also constructed pGIPZ Lentiviral shRNA inserted with previously reported si*Pkd1*^3297^ sequences 5′-CATGTGAGCAACATCACC-3′ (ref. 67) using *Xho*I and *Mlu*I. These *Pkd1* shRNAs showed up to ~90% knockdown of PC1 (Supplementary Fig. 2a,b). For PC2 knockdown, we used pGIPZ Lentiviral shRNA containing following antisense sequences 5′-TTTCCAATATCTCTTCCAC-3′. For Rabep1, GGA1 and Arl3 inhibition, we used three shRNAs for Rabep1, four shRNAs for GGA1 and two shRNAs for Arl3. Rabep1 antisense #1; 5′- TAAATTCTTGCTCAAGCTG-3′, Rabep1 antisense #2; 5′-TCATCTATAGCTTCTTGCT-3′, Rabep1 antisense #3; 5′-TGTTGTTCATCCTCCTCCT-3′, GGA1 antisense #1; 5′-TTGATGAGCTTATTGGCAG-3′, GGA1 antisense #2; 5′-TGTACAGGTTGATCACCTG-3′, GGA1 antisense #3; 5′-ACAGGTTGATCACCTGGGT-3′, GGA1 antisense #4; 5′-TCAGCTCTGTGACCACAGG-3′, Arl3 antisense #1; 5′-TTCTTCCTCCAGTAATTCC-3′, Arl3 antisense #2; 5′-AACTTCTCCAGTATGGTCT-3′. pGIPZ Lentiviral vector containing gene specific shRNA was transfected into 293T cells with viral packaging plasmids, and the resulting lentivirus were produced and used according to the manufacturer’s instruction.

### Yeast two-hybrid screening

The human PKD1 cDNA (Accession L33243) fragment corresponding to bp 12,473–13,120 encoding the C-terminal 215 amino acids was isolated by PCR and subcloned into the *Sal*I and *Not*I sites of the pPC97 vector in-frame with the GAL4 DNA-binding domain^68^. YRG-2 yeast cells (Stratagene) were transformed with bait and subsequently with an adult rat hippocampus random primed cDNA library cloned into pPC86. The protocols used in this yeast two-hybrid system were performed as described previously^22^. In brief, positive yeast clones were selected on triple-deficient plates (Leu-, Trp-, and His-) and confirmed by positive β-gal activity. The pPC86-based prey plasmids were recovered and co-transformed into YRG-2 with either the bait vector or the original pPC97 vector to confirm the interaction.

### Statistical analysis

Statistical analysis was performed by ANOVA to calculate statistical significance between more than two experimental groups. Student’s *t*-test was used for the statistical significance between two experimental groups. All data are presented as mean±s.e.m.

## Additional information

**How to cite this article:** Kim, H. *et al.* Ciliary membrane proteins traffic through the Golgi via a Rabep1/GGA1/Arl3-dependent mechanism. *Nat. Commun.* 5:5482 doi: 10.1038/ncomms6482 (2014).

## Supplementary Material

Supplementary Information Supplementary Figures 1-5.

## Figures and Tables

**Table 1:** Stable MDCK cells with tetracycline-inducible expression of mouse PC1 proteins.

**MDCK cells**	**PC1 variant expressed**	**Description**
MDCK^PC1WT^	PC1 ^WT^	Full-length wild-type PC1 with a C-terminal FLAG tag
MDCK^PC1-2M^	PC1 ^2M^	Full-length PC1 with a C-terminal FLAG tag, containing amino-acid substitutions at L4219P and A4222P within the coiled-coil domain
MDCK^PC1-R/X^	PC1 ^R/X^	PC1 terminating at R4218X with a C-terminal FLAG tag before the stop codon
MDCK^PC1V^	PC1 ^V^	Full-length PC1 with a C-terminal FLAG tag, containing amino-acid substitution T3041V at GPS cleavage site

## References

[b1] Singla V. & Reiter J. F. The primary cilium as the cell's antenna: signaling at a sensory organelle. Science 313, 629–633 (2006). 16888132 10.1126/science.1124534

[b2] Nauli S. M. *et al.* Polycystins 1 and 2 mediate mechanosensation in the primary cilium of kidney cells. Nat. Genet. 33, 129–137 (2003). 12514735 10.1038/ng1076

[b3] Pazour G. J. Intraflagellar transport and cilia-dependent renal disease: the ciliary hypothesis of polycystic kidney disease. J. Am. Soc. Nephrol. 15, 2528–2536 (2004). 15466257 10.1097/01.ASN.0000141055.57643.E0

[b4] Hu Q. *et al.* A septin diffusion barrier at the base of the primary cilium maintains ciliary membrane protein distribution. Science 329, 436–439 (2010). 20558667 10.1126/science.1191054 PMC3092790

[b5] Nachury M. V., Seeley E. S. & Jin H. Trafficking to the ciliary membrane: how to get across the periciliary diffusion barrier? Annu. Rev. Cell Dev. Biol. 26, 59–87 (2010). 19575670 10.1146/annurev.cellbio.042308.113337 PMC2952038

[b6] Pedersen L. B. & Rosenbaum J. L. Intraflagellar transport (IFT) role in ciliary assembly, resorption and signalling. Curr. Top. Dev. Biol. 85, 23–61 (2008). 19147001 10.1016/S0070-2153(08)00802-8

[b7] Consortium TEPKD. The polycystic kidney disease 1 gene encodes a 14 kb transcript and lies within a duplicated region on chromosome 16. Cell 77, 881–894 (1994). 8004675 10.1016/0092-8674(94)90137-6

[b8] Mochizuki T. *et al.* PKD2, a gene for polycystic kidney disease that encodes an integral membrane protein. Science 272, 1339–1342 (1996). 8650545 10.1126/science.272.5266.1339

[b9] Pazour G. J., San Agustin J. T., Follit J. A., Rosenbaum J. L. & Witman G. B. Polycystin-2 localizes to kidney cilia and the ciliary level is elevated in orpk mice with polycystic kidney disease. Curr. Biol. 12, R378–R380 (2002). 12062067 10.1016/s0960-9822(02)00877-1

[b10] Yoder B. K., Hou X. & Guay-Woodford L. M. The polycystic kidney disease proteins, polycystin-1, polycystin-2, polaris, and cystin, are co-localized in renal cilia. J. Am. Soc. Nephrol. 13, 2508–2516 (2002). 12239239 10.1097/01.asn.0000029587.47950.25

[b11] Praetorius H. A. & Spring K. R. Bending the MDCK cell primary cilium increases intracellular calcium. J. Membr. Biol. 184, 71–79 (2001). 11687880 10.1007/s00232-001-0075-4

[b12] Hughes J. *et al.* The polycystic kidney disease 1 (PKD1) gene encodes a novel protein with multiple cell recognition domains. Nat. Genet. 10, 151–160 (1995). 7663510 10.1038/ng0695-151

[b13] Nims N., Vassmer D. & Maser R. L. Transmembrane domain analysis of polycystin-1, the product of the polycystic kidney disease-1 (PKD1) gene: evidence for 11 membrane-spanning domains. Biochemistry 42, 13035–13048 (2003). 14596619 10.1021/bi035074c

[b14] Qian F. *et al.* Cleavage of polycystin-1 requires the receptor for egg jelly domain and is disrupted by human autosomal-dominant polycystic kidney disease 1-associated mutations. Proc. Natl Acad. Sci. USA 99, 16981–16986 (2002). 12482949 10.1073/pnas.252484899 PMC139255

[b15] Yu S. *et al.* Essential role of cleavage of Polycystin-1 at G protein-coupled receptor proteolytic site for kidney tubular structure. Proc. Natl Acad. Sci. USA 104, 18688–18693 (2007). 18003909 10.1073/pnas.0708217104 PMC2141838

[b16] Wei W., Hackmann K., Xu H., Germino G. & Qian F. Characterization of cis-autoproteolysis of polycystin-1, the product of human polycystic kidney disease 1 gene. J. Biol. Chem. 282, 21729–21737 (2007). 17525154 10.1074/jbc.M703218200

[b17] Castelli M. *et al.* Polycystin-1 binds Par3/aPKC and controls convergent extension during renal tubular morphogenesis. Nat. Commun. 4, 2658 (2013). 24153433 10.1038/ncomms3658 PMC3967097

[b18] Kurbegovic A. *et al.* Novel functional complexity of polycystin-1 by GPS cleavage in vivo: role in polycystic kidney disease. Mol. Cell Biol. 34, 3341–3353 (2014). 24958103 10.1128/MCB.00687-14 PMC4135549

[b19] Arac D. *et al.* A novel evolutionarily conserved domain of cell-adhesion GPCRs mediates autoproteolysis. EMBO J. 31, 1364–1378 (2012). 22333914 10.1038/emboj.2012.26 PMC3321182

[b20] Cai Y. *et al.* Identification and characterization of polycystin-2, the PKD2 gene product. J. Biol. Chem. 274, 28557–28565 (1999). 10497221 10.1074/jbc.274.40.28557

[b21] Koulen P. *et al.* Polycystin-2 is an intracellular calcium release channel. Nat. Cell Biol. 4, 191–197 (2002). 11854751 10.1038/ncb754

[b22] Qian F. *et al.* PKD1 interacts with PKD2 through a probable coiled-coil domain. Nat. Genet. 16, 179–183 (1997). 9171830 10.1038/ng0697-179

[b23] Tsiokas L., Kim E., Arnould T., Sukhatme V. P. & Walz G. Homo- and heterodimeric interactions between the gene products of PKD1 and PKD2. Proc. Natl Acad. Sci. USA 94, 6965–6970 (1997). 9192675 10.1073/pnas.94.13.6965 PMC21268

[b24] Zhu J. *et al.* Structural model of the TRPP2/PKD1 C-terminal coiled-coil complex produced by a combined computational and experimental approach. Proc. Natl Acad. Sci. USA 108, 10133–10138 (2011). 21642537 10.1073/pnas.1017669108 PMC3121833

[b25] Chapin H. C., Rajendran V. & Caplan M. J. Polycystin-1 surface localization is stimulated by polycystin-2 and cleavage at the G protein-coupled receptor proteolytic site. Mol. Biol. Cell 21, 4338–4348 (2010). 20980620 10.1091/mbc.E10-05-0407 PMC3002387

[b26] Grimm D. H. *et al.* Polycystin-1 distribution is modulated by polycystin-2 expression in mammalian cells. J. Biol. Chem. 278, 36786–36793 (2003). 12840011 10.1074/jbc.M306536200

[b27] Hanaoka K. *et al.* Co-assembly of polycystin-1 and -2 produces unique cation-permeable currents. Nature 408, 990–994 (2000). 11140688 10.1038/35050128

[b28] Geng L. *et al.* Polycystin-2 traffics to cilia independently of polycystin-1 by using an N-terminal RVxP motif. J. Cell Sci. 119, 1383–1395 (2006). 16537653 10.1242/jcs.02818

[b29] Ward H. H. *et al.* A conserved signal and GTPase complex are required for the ciliary transport of polycystin-1. Mol. Biol. Cell 22, 3289–3305 (2011). 21775626 10.1091/mbc.E11-01-0082 PMC3172256

[b30] Hoffmeister H. *et al.* Polycystin-2 takes different routes to the somatic and ciliary plasma membrane. J. Cell Biol. 192, 631–645 (2011). 21321097 10.1083/jcb.201007050 PMC3044124

[b31] Xu C. *et al.* Human ADPKD primary cyst epithelial cells with a novel, single codon deletion in the PKD1 gene exhibit defective ciliary polycystin localization and loss of flow-induced Ca2+ signaling. Am. J. Physiol. Renal. Physiol. 292, F930–F945 (2007). 17090781 10.1152/ajprenal.00285.2006 PMC3586432

[b32] Freedman B. S. *et al.* Reduced ciliary polycystin-2 in induced pluripotent stem cells from polycystic kidney disease patients with PKD1 mutations. J. Am. Soc. Nephrol. 24, 1571–1586 (2013). 24009235 10.1681/ASN.2012111089 PMC3785271

[b33] Piontek K., Menezes L. F., Garcia-Gonzalez M. A., Huso D. L. & Germino G. G. A critical developmental switch defines the kinetics of kidney cyst formation after loss of Pkd1. Nat. Med. 13, 1490–1495 (2007). 17965720 10.1038/nm1675 PMC2302790

[b34] Cebotaru V. *et al.* Polycystin-1 negatively regulates Polycystin-2 expression via the aggresome/autophagosome pathway. J. Biol. Chem. 289, 6404–6414 (2014). 24459142 10.1074/jbc.M113.501205 PMC3945307

[b35] Freeze H. H. in *Current Protocols* *in Cell Biology* (eds J. S. Bonifacino *et al.*) chapter 15, 12 (John Wiley and Sons, Inc., 2001).

[b36] Overgaard C. E. *et al.* Deciliation is associated with dramatic remodeling of epithelial cell junctions and surface domains. Mol. Biol. Cell 20, 102–113 (2009). 19005211 10.1091/mbc.E08-07-0741 PMC2613083

[b37] Pisitkun T., Shen R. F. & Knepper M. A. Identification and proteomic profiling of exosomes in human urine. Proc. Natl Acad. Sci. USA 101, 13368–13373 (2004). 15326289 10.1073/pnas.0403453101 PMC516573

[b38] Webb C. P., Lane K., Dawson A. P., Vande Woude G. F. & Warn R. M. C-Met signalling in an HGF/SF-insensitive variant MDCK cell line with constitutive motile/invasive behaviour. J. Cell Sci. 109, (Pt 9): 2371–2381 (1996). 8886986 10.1242/jcs.109.9.2371

[b39] Shalamanova L., Kubler B., Scharf J. G. & Braulke T. MDCK cells secrete neutral proteases cleaving insulin-like growth factor-binding protein-2 to -6. Am. J. Physiol. Endocrinol. Metab. 281, E1221–E1229 (2001). 11701437 10.1152/ajpendo.2001.281.6.E1221

[b40] Stenmark H., Vitale G., Ullrich O. & Zerial M. Rabaptin-5 is a direct effector of the small GTPase Rab5 in endocytic membrane fusion. Cell 83, 423–432 (1995). 8521472 10.1016/0092-8674(95)90120-5

[b41] Wodarczyk C. *et al.* A novel mouse model reveals that polycystin-1 deficiency in ependyma and choroid plexus results in dysfunctional cilia and hydrocephalus. PLoS ONE 4, e7137 (2009). 19774080 10.1371/journal.pone.0007137 PMC2743994

[b42] Boman A. L., Zhang C., Zhu X. & Kahn R. A. A family of ADP-ribosylation factor effectors that can alter membrane transport through the trans-Golgi. Mol. Biol. Cell 11, 1241–1255 (2000). 10749927 10.1091/mbc.11.4.1241 PMC14844

[b43] Bonifacino J. S. The GGA proteins: adaptors on the move. Nat. Rev. Mol. Cell Biol. 5, 23–32 (2004). 14708007 10.1038/nrm1279

[b44] Puertollano R., Randazzo P. A., Presley J. F., Hartnell L. M. & Bonifacino J. S. The GGAs promote ARF-dependent recruitment of clathrin to the TGN. Cell 105, 93–102 (2001). 11301005 10.1016/s0092-8674(01)00299-9

[b45] Mattera R., Arighi C. N., Lodge R., Zerial M. & Bonifacino J. S. Divalent interaction of the GGAs with the Rabaptin-5-Rabex-5 complex. EMBO J. 22, 78–88 (2003). 12505986 10.1093/emboj/cdg015 PMC140067

[b46] Kahn R. A. *et al.* Arf family GTPases: roles in membrane traffic and microtubule dynamics. Biochem. Soc. Trans. 33, 1269–1272 (2005). 16246095 10.1042/BST0331269

[b47] Dell'Angelica E. C. *et al.* GGAs: a family of ADP ribosylation factor-binding proteins related to adaptors and associated with the Golgi complex. J. Cell Biol. 149, 81–94 (2000). 10747089 10.1083/jcb.149.1.81 PMC2175099

[b48] Wang J. & Deretic D. Molecular complexes that direct rhodopsin transport to primary cilia. Prog. Retin. Eye Res. 38, 1–19 (2014). 24135424 10.1016/j.preteyeres.2013.08.004 PMC3883129

[b49] Van Valkenburgh H., Shern J. F., Sharer J. D., Zhu X. & Kahn R. A. ADP-ribosylation factors (ARFs) and ARF-like 1 (ARL1) have both specific and shared effectors: characterizing ARL1-binding proteins. J. Biol. Chem. 276, 22826–22837 (2001). 11303027 10.1074/jbc.M102359200

[b50] Zhou C., Cunningham L., Marcus A. I., Li Y. & Kahn R. A. Arl2 and Arl3 regulate different microtubule-dependent processes. Mol. Biol. Cell 17, 2476–2487 (2006). 16525022 10.1091/mbc.E05-10-0929 PMC1446103

[b51] Schrick J. J., Vogel P., Abuin A., Hampton B. & Rice D. S. ADP-ribosylation factor-like 3 is involved in kidney and photoreceptor development. Am. J. Pathol. 168, 1288–1298 (2006). 16565502 10.2353/ajpath.2006.050941 PMC1606550

[b52] Kottgen M. *et al.* Trafficking of TRPP2 by PACS proteins represents a novel mechanism of ion channel regulation. EMBO J. 24, 705–716 (2005). 15692563 10.1038/sj.emboj.7600566 PMC549624

[b53] Fedeles S. V. *et al.* A genetic interaction network of five genes for human polycystic kidney and liver diseases defines polycystin-1 as the central determinant of cyst formation. Nat. Genet. 43, 639–647 (2011). 21685914 10.1038/ng.860 PMC3547075

[b54] Hopp K. *et al.* Functional polycystin-1 dosage governs autosomal dominant polycystic kidney disease severity. J. Clin. Invest. 122, 4257–4273 (2012). 23064367 10.1172/JCI64313 PMC3484456

[b55] Margeta-Mitrovic M., Jan Y. N. & Jan L. Y. A trafficking checkpoint controls GABA(B) receptor heterodimerization. Neuron 27, 97–106 (2000). 10939334 10.1016/s0896-6273(00)00012-x

[b56] Karner C. M. *et al.* Wnt9b signaling regulates planar cell polarity and kidney tubule morphogenesis. Nat. Genet. 41, 793–799 (2009). 19543268 10.1038/ng.400 PMC2761080

[b57] Emmer B. T., Maric D. & Engman D. M. Molecular mechanisms of protein and lipid targeting to ciliary membranes. J. Cell Sci. 123, 529–536 (2010). 20145001 10.1242/jcs.062968 PMC2818192

[b58] Li Y., Wei Q., Zhang Y., Ling K. & Hu J. The small GTPases ARL-13 and ARL-3 coordinate intraflagellar transport and ciliogenesis. J. Cell Biol. 189, 1039–1051 (2010). 20530210 10.1083/jcb.200912001 PMC2886347

[b59] Tai A. W., Chuang J. Z., Bode C., Wolfrum U. & Sung C. H. Rhodopsin's carboxy-terminal cytoplasmic tail acts as a membrane receptor for cytoplasmic dynein by binding to the dynein light chain Tctex-1. Cell 97, 877–887 (1999). 10399916 10.1016/s0092-8674(00)80800-4

[b60] Omori Y. *et al.* Elipsa is an early determinant of ciliogenesis that links the IFT particle to membrane-associated small GTPase Rab8. Nat. Cell Biol. 10, 437–444 (2008). 18364699 10.1038/ncb1706

[b61] Ismail S. A. *et al.* Structural basis for Arl3-specific release of myristoylated ciliary cargo from UNC119. EMBO J. 31, 4085–4094 (2012). 22960633 10.1038/emboj.2012.257 PMC3474929

[b62] Wright K. J. *et al.* An ARL3-UNC119-RP2 GTPase cycle targets myristoylated NPHP3 to the primary cilium. Genes Dev. 25, 2347–2360 (2011). 22085962 10.1101/gad.173443.111 PMC3222901

[b63] Fogelgren B. *et al.* The exocyst protein Sec10 interacts with Polycystin-2 and knockdown causes PKD-phenotypes. PLoS Genet. 7, e1001361 (2011). 21490950 10.1371/journal.pgen.1001361 PMC3072367

[b64] Su X. *et al.* Bardet-Biedl syndrome proteins 1 and 3 regulate the ciliary trafficking of polycystic kidney disease 1 protein. Hum. Mol. Genet. 23, 5441–5451 (2014). 24939912 10.1093/hmg/ddu267 PMC4168828

[b65] Sang L. *et al.* Mapping the NPHP-JBTS-MKS protein network reveals ciliopathy disease genes and pathways. Cell 145, 513–528 (2011). 21565611 10.1016/j.cell.2011.04.019 PMC3383065

[b66] Woodward O. M. *et al.* Identification of a polycystin-1 cleavage product, P100, that regulates store operated Ca entry through interactions with STIM1. PLoS ONE 5, e12305 (2010). 20808796 10.1371/journal.pone.0012305 PMC2925899

[b67] Battini L. *et al.* Loss of polycystin-1 causes centrosome amplification and genomic instability. Hum. Mol. Genet. 17, 2819–2833 (2008). 18566106 10.1093/hmg/ddn180 PMC2722891

[b68] Fields S. & Sternglanz R. The two-hybrid system: an assay for protein-protein interactions. Trends Genet. 10, 286–292 (1994). 7940758 10.1016/0168-9525(90)90012-u

[b69] Lupas A., Van Dyke M. & Stock J. Predicting coiled coils from protein sequences. Science 252, 1162–1164 (1991). 2031185 10.1126/science.252.5009.1162

[b70] Qian F., Watnick T. J., Onuchic L. F. & Germino G. G. The molecular basis of focal cyst formation in human autosomal dominant polycystic kidney disease type I. Cell 87, 979–987 (1996). 8978603 10.1016/s0092-8674(00)81793-6

