# Supplementary material for: Ciliary membrane proteins traffic through the Golgi via a Rabep1/GGA1/Arl3-dependent mechanism
Source: Nat Commun. 2014 Nov 18;5:5482. doi: 10.1038/ncomms6482 (PMC4237283; doi:10.1038/ncomms6482)
Supplement: Supplementary Information — Supplementary Figures 1-5. [file ncomms6482-s1.pdf]

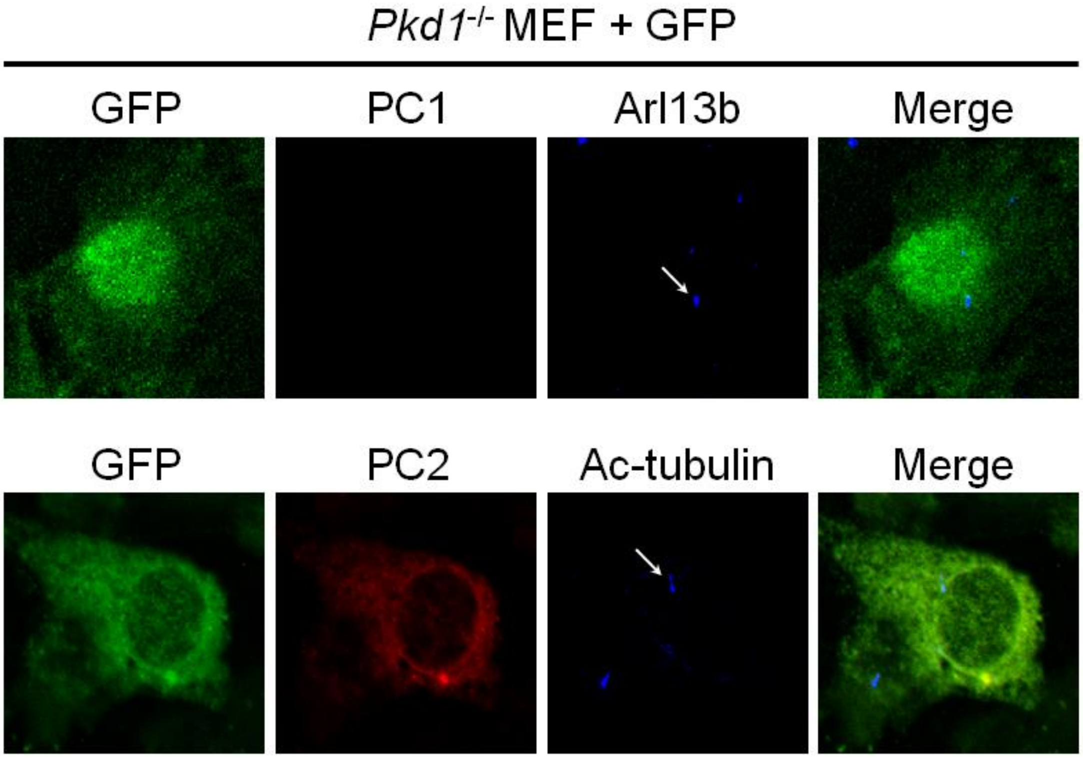

Supplementary Figure 1 Exogenously expressed GFP does not rescue ciliary localization of PC2 in *Pkd1*$^{-/-}$ MEFs. The cells transfected with a GFP expression plasmid were stained with PC1(E4) (upper panel) or PC2 (lower panel). Arl13b or Ac-tubulin was used as a ciliary marker. Cilia are indicated by an arrow.

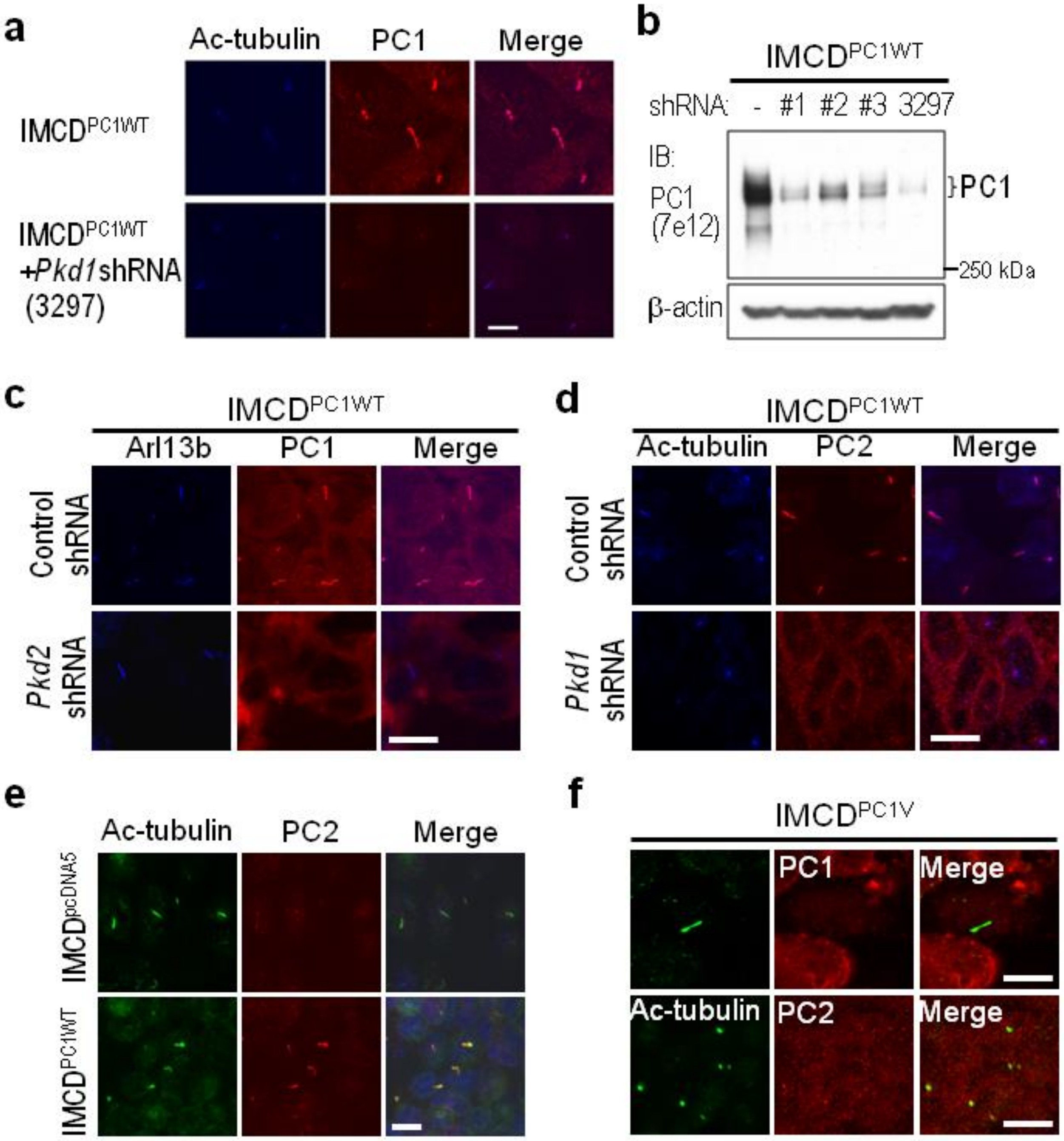

a
Ac-tubulin
PC1
Merge
IMCD^{PC1WT}
IMCD^{PC1WT}
+*Pkd1* shRNA
(3297)
b
IMCD^{PC1WT}
shRNA: - #1 #2 #3 3297
IB:
PC1
(7e12)
}PC1
250 kDa
β-actin
c
IMCD^{PC1WT}
Arl13b
PC1
Merge
Control shRNA
Pkd2 shRNA
d
IMCD^{PC1WT}
Ac-tubulin
PC2
Merge
Control shRNA
Pkd1 shRNA
e
Ac-tubulin
PC2
Merge
IMCD^{pcDNA5}
IMCD^{PC1WT}
f
IMCD^{PC1V}
PC1
Merge
Ac-tubulin
PC2
Merge

Supplementary Figure 2 Interdependence of PC1 and PC2 ciliary localization in IMCD cells (**a**-**e**). (**a**) Confocal images of IMCD cells with stable expression of full-length C-terminal FLAG-tagged mouse PC1 ($IMCD^{PC1WT}$, upper panel) and $IMCD^{PC1WT}$ expressing *Pkd1* shRNA 3297[67] (lower panel). Cells were stained with antibodies against PC1(E4) (red) and acetylated (Ac) tubulin (blue). (**b**) Western blot analysis (at right) shows knockdown of PC1 expression in MCD^{PC1WT} cells by various *Pkd1* shRNAs described in Method. (**c**) Confocal images of $IMCD^{PC1WT}$ cells expressing control (upper panel) or *Pkd2* (lower panel) shRNA. Cells were stained with antibodies against PC1(E4) (red) and Arl13b (blue). Note the absence of ciliary staining of PC1 by *Pkd2* shRNA. (**d**) Confocal images of $IMCD^{PC1WT}$ cells expressing control (upper panel) or *Pkd1* (lower panel) shRNA. Cells were stained with antibodies against PC2 (red) and Ac-tubulin (blue). Note the absence of ciliary staining of PC2 by *Pkd1* shRNA. (**e**) Confocal images of $IMCD^{pcDNA5}$ cells (empty vector control cells) and MCD^{PC1WT} cells stained with antibodies against PC2 (red) and Ac-tubulin (green). Note that $IMCD^{PC1WT}$ cells displays increased PC2 ciliary signal intensity compared to $IMCD^{pcDNA5}$ cells. (**f**) Confocal images of $IMCD^{PC1V}$ cells with stable expression of recombinant non-cleavable $PC1^{V}$ showing no ciliary staining of $PC1^{V}$ and endogenous PC2. Scale bar, 10 μm.

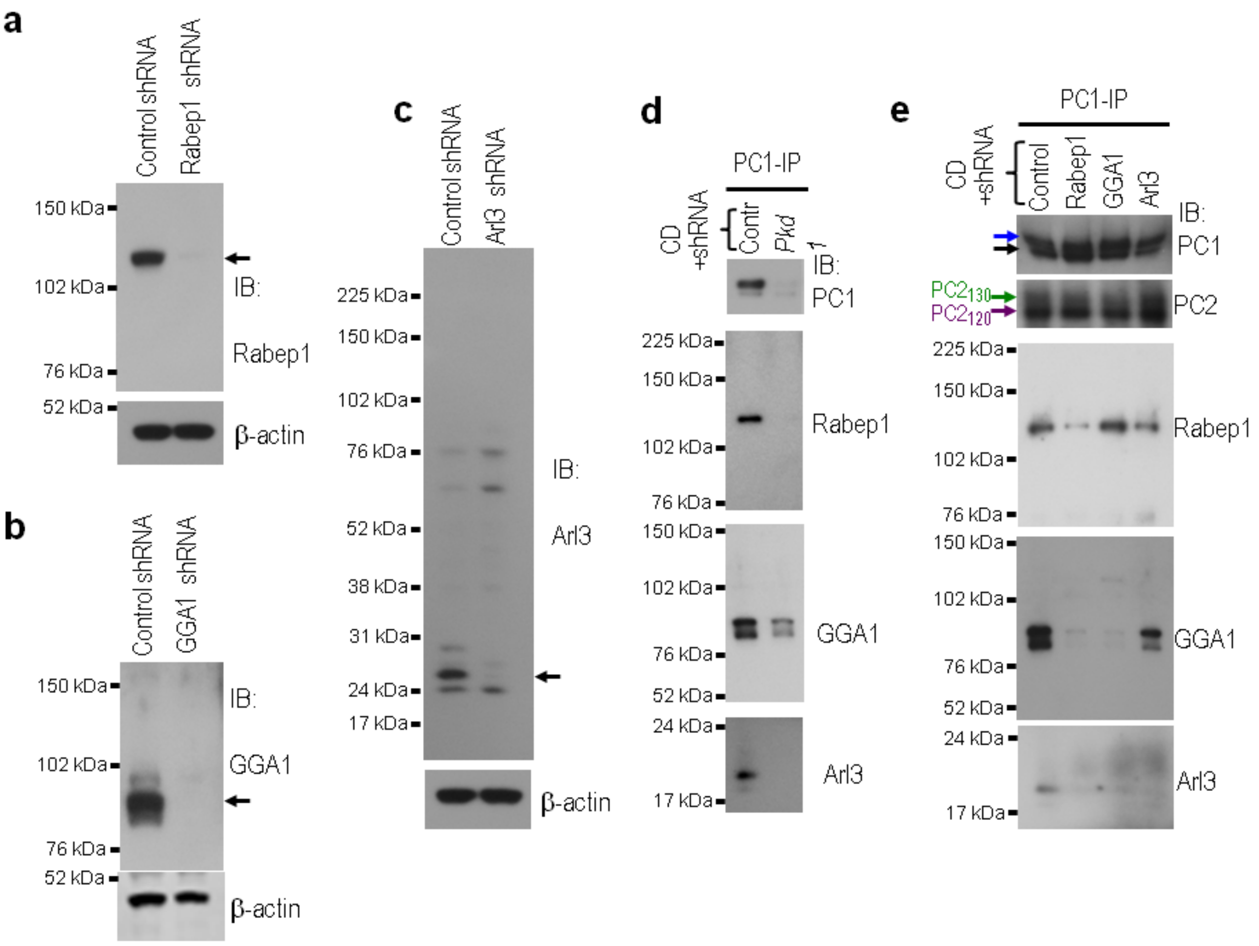

Supplementary Figure 3 Western blots showing effective knockdown of Rabep1 (**a**), GGA1 (**b**) or Arl3 (**c**). The specific protein bands are indicated by an arrow. The molecular weight markers are shown on left. (**d**) The larger areas of the blots for Fig. 7a. (**e**) The larger areas of the blots for Fig. 7f.

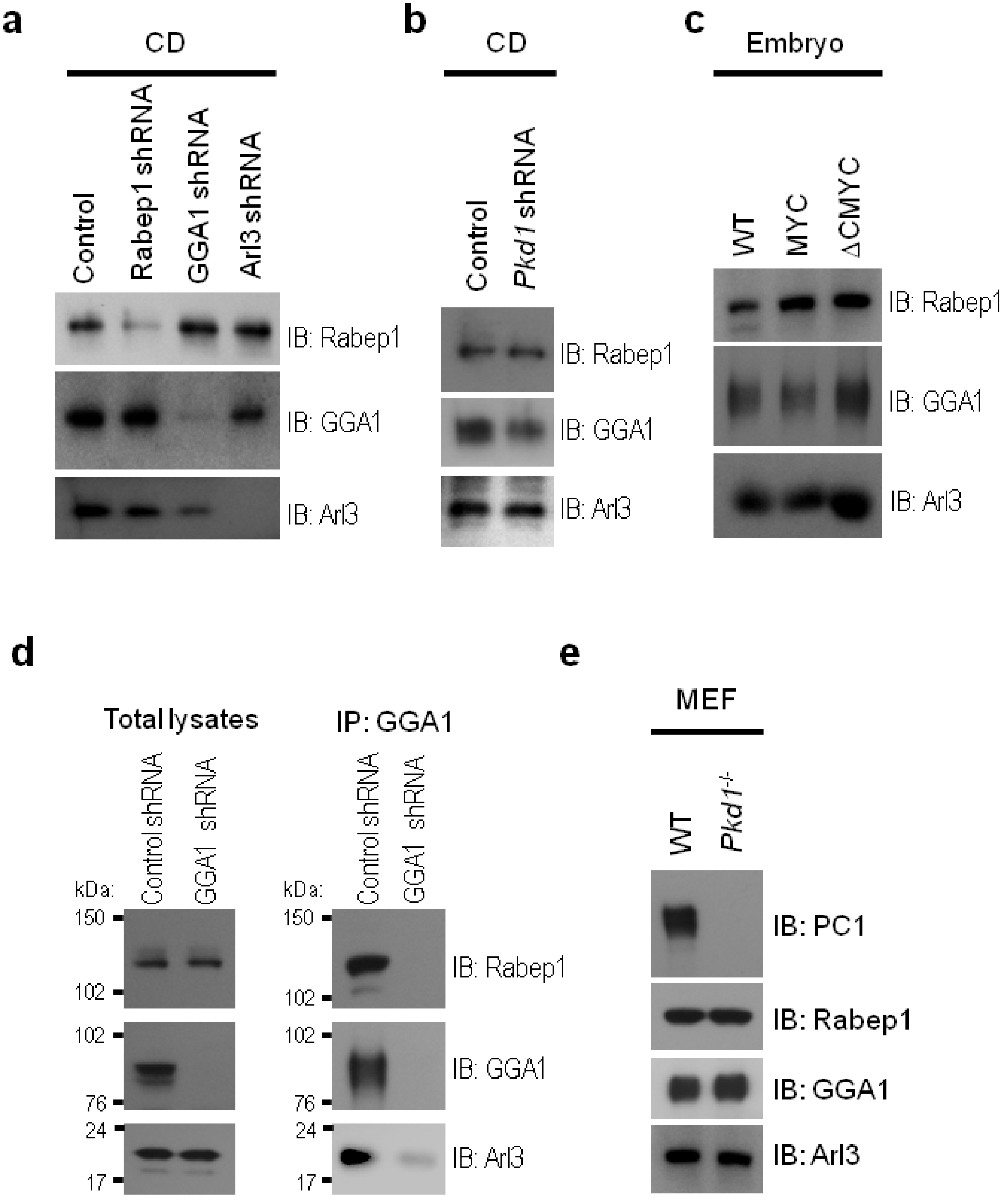
a
CD
Control
Rabep1 shRNA
GGA1 shRNA
Arl3 shRNA
IB: Rabep1
IB: GGA1
IB: Arl3
b
CD
Control
Pkd1 shRNA
IB: Rabep1
IB: GGA1
IB: Arl3
c
Embryo
WT
MYC
ΔCMYC
IB: Rabep1
IB: GGA1
IB: Arl3
d
Total lysates
Control shRNA
GGA1 shRNA
kDa:
150
102
102
76
24
17
IP: GGA1
Control shRNA
GGA1 shRNA
kDa:
150
102
102
76
24
17
IB: Rabep1
IB: GGA1
IB: Arl3
e
MEF
WT
Pkd1-/-
IB: PC1
IB: Rabep1
IB: GGA1
IB: Arl3

Supplementary Figure 4 Western blots of various total lysates probed with anti-Rabep1, anti-GGA1 or anti-Arl3. (**a**) Validation of lentiviral shRNA-mediated silencing of the respective genes in CD cells used in Fig. 7f and g. (**b**) Similar levels of Rabep1, GGA1 or Arl3 in CD cells with or without *Pkd1* knockdown used for co-IP experiment in Fig. 7a. (**c**) Similar levels of Rabep1, GGA1 or Arl3 in wild-type (WT), *Pkd1*$^{\mathrm{MYC/MYC}}$ (MYC) and *Pkd1*$^{\Delta\mathrm{CMYC}/\Delta\mathrm{CMYC}}$ (ΔCMYC) embryos used for co-IP experiment in Fig. 7b. (**d**) Specificity of co-immunoprecipitation of native GGA1 and Arl3 by anti-GGA1 shown in Fig. 7g is validated using CD cells with GGA1 knockdown. (**e**) Similar levels of Rabep1, GGA1 or Arl3 in wild-type (WT) and *Pkd1*$^{-/-}$ MEFs used for co-IP experiment in Fig. 7h.

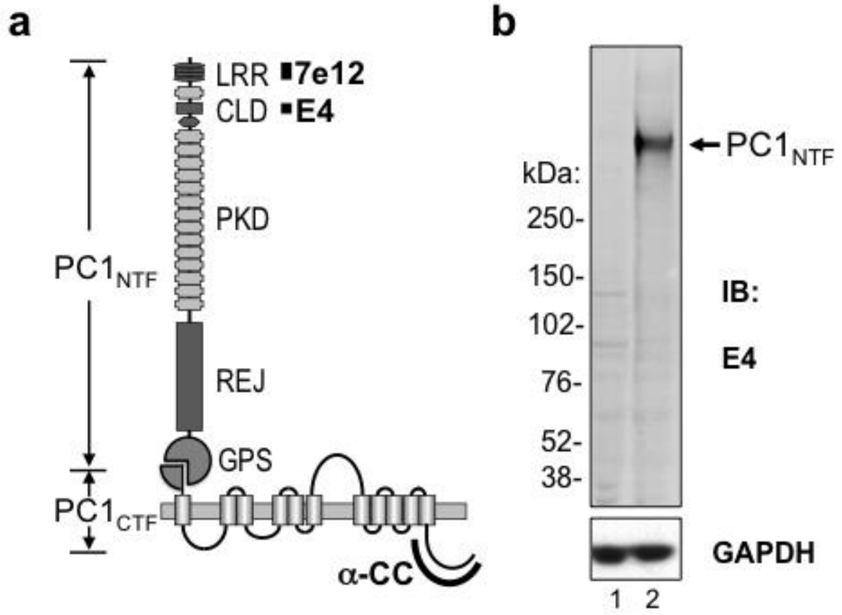

Supplementary Figure 5 Specificity of PC1 E4 mouse monoclonal antibody. (**a**) A schematic diagram of the domain organization of PC1. PKD: PKD repeats; CLD, C-type lectin binding domain. GPS: G-protein coupled receptor proteolytic cleavage site. E4 monoclonal antibody against PC1 was generated from mice immunized with recombinant 130 aa polypeptide corresponding to residues 406-535 (CLD) of human PC1 protein, as indicated by a black bar. The positions of epitopes recognized by the other antibodies (7e12, α-CC) used in this study are also indicated. (**b**) Western blots demonstrating the specificity of E4 antibody. E4 detects mouse $PC1_{NTF}$ as indicated from lysate of HEK cells with stable expression of mouse full-length *Pkd1* cDNA (lane 2), but not from that of HEK cells containing empty vector (lane 1). The molecular weight marker is shown on left. Western blot with anti-GAPDH shows equal loading.
